# Anterior and Posterior Left Inferior Frontal Gyrus Contribute to the Implementation of Grammatical Determiners During Language Production

**DOI:** 10.3389/fpsyg.2020.00685

**Published:** 2020-04-27

**Authors:** Byurakn Ishkhanyan, Violaine Michel Lange, Kasper Boye, Jesper Mogensen, Anke Karabanov, Gesa Hartwigsen, Hartwig Roman Siebner

**Affiliations:** ^1^Department of Nordic Studies and Linguistics, University of Copenhagen, Copenhagen, Denmark; ^2^Danish Research Centre for Magnetic Resonance, Copenhagen University Hospital Hvidovre, Hvidovre, Denmark; ^3^Department of Linguistics, Cognitive Science and Semiotics, Aarhus University, Aarhus, Denmark; ^4^The Unit for Cognitive Neuroscience (UCN), Department of Psychology, University of Copenhagen, Copenhagen, Denmark; ^5^Lise Meitner Research Group Cognition and Plasticity, Max Planck Institute for Human Cognitive and Brain Sciences Leipzig, Leipzig, Germany; ^6^Faculty of Medical and Health Sciences, Institute for Clinical Medicine, University of Copenhagen, Copenhagen, Denmark; ^7^Department of Neurology, Copenhagen University Hospital Bispebjerg, Copenhagen, Denmark

**Keywords:** language production, Broca’s area, grammar, lexicon, interior frontal gyrus

## Abstract

The left inferior frontal gyrus (IFG) is a key region for language comprehension and production. Previous studies point to a preferential involvement of left anterior IFG (aIFG) in lexical and semantic processes, while the posterior IFG (pIFG) has been implicated in supporting syntactic and phonological processes. Here we used focal neuronavigated transcranial magnetic stimulation (TMS) to probe the functional involvement of left IFG in lexical and grammatical processing at the sentence level. We applied 10 Hz TMS effective or sham bursts to left aIFG and pIFG, while healthy volunteers performed an adjective-noun production task contrasting grammatical and lexical determiners. For each trial, we measured the time from the stimulus onset to the moment of articulation (response time) and the time from articulation onset to the end of articulation (duration). Focal TMS of IFG generally delayed response times. The TMS-induced delay in response times was relatively stronger for the grammatical condition compared to the lexical condition, when TMS targeted aIFG. Articulation of the determiner was generally shorter in trials presenting grammatical determiners relative to lexical determiners. The shorter articulation time for grammar determiners was facilitated by effective TMS to pIFG. Together, the effects of TMS on task performance provide novel evidence for a joint involvement of anterior and posterior parts of left IFG in implementing grammatical determiners during language production, suggesting an involvement of aIFG in the initiation and pIFG in the production of grammatically appropriate verbal responses at the sentence level.

## Introduction

The left inferior frontal gyrus (IFG) is a key node of the neural networks engaged in language processing in the human brain and has been subdivided in several areas crucial for different aspects of language processing. Neuroimaging studies have shown that the anterior part of the left IFG [aIFG, pars orbitalis, Brodmann’s area (BA) 47, and pars triangularis, BA 45] is associated with semantic processing both during language comprehension and production while the posterior part (pIFG; pars opercularis BA 44) is engaged in syntactic aspects of language comprehension and production (Caplan et al., 1998; Dapretto and Bookheimer, 1999; Crosson et al., 2001; Gaillard et al., 2003; Hagoort et al., 2004; Haller et al., 2005; Friederici, 2009; Heim et al., 2009a). However, most of the previous work focused on the distinction between BA 44 and BA 45 and some studies reported increased BA 45 activation across both syntactic and semantic tasks (Dapretto and Bookheimer, 1999; Friederici et al., 2000; Haller et al., 2005). Consequently, Hagoort (2005) suggested a unification gradient in the left IFG, according to which the more anterior parts (BA 47 and the anterior part of BA 45) are mainly involved in semantic processing, whereas more posterior parts (the posterior part of BA 45 and BA 44) contribute to syntactic processing. Parts of the left pIFG have also been associated with various phonological tasks (e.g., Chee et al., 1999; Poldrack et al., 1999; Hagoort, 2005). Damage to the left IFG can cause severe language deficits and is often associated with Broca’s aphasia (e.g., Druks, 2016), although Broca’s aphasia does not necessarily include a lesion of the IFG (Dronkers et al., 2004; Fridriksson et al., 2018) and damage to Broca’s area alone is not sufficient to produce Broca’s aphasia (Dronkers, 2000; Mohr, 1976).

Neuroimaging studies have been complemented by transcranial magnetic stimulation (TMS) studies, providing evidence for a functional-anatomical double dissociation within the left IFG: perturbation of the aIFG affects semantic word decisions (Devlin et al., 2003; Zhu et al., 2015), while perturbation of the pIFG impairs phonological decisions and phonological working memory (Nixon et al., 2004; Gough et al., 2005; Hartwigsen et al., 2010a, b, 2015; Karabanov et al., 2015). Other studies further demonstrated a key role of left pIFG in picture naming and syntactic processing (Schuhmann et al., 2011; Acheson and Hagoort, 2013; Krieger-Redwood and Jefferies, 2014; Kuhnke et al., 2017).

While the above-mentioned TMS studies mainly focused on single word processing, a series of electroencephalography (EEG) and magnetoencephalography (MEG) studies used multi-word production paradigms to explore the time course and the order in which the components of a phrase are being planned (Eulitz et al., 2000; Habets et al., 2008; Bürki and Laganaro, 2014; Pylkkänen et al., 2014; Michel Lange et al., 2015; Bürki et al., 2016). While Eulitz et al. (2000) did not detect an EEG signal difference between adjective-noun and isolated noun naming in German, Michel Lange et al. (2015) showed that adjective-noun phrases take longer to plan than isolated nouns in French. Using English stimuli, Pylkkänen et al. (2014) also identified a difference between isolated noun phrase (e.g., cat) and adjective-noun phrase (e.g., black cat) production. A similar temporal difference was observed by Bürki and Laganaro (2014) using determiner-adjective-noun phrases (the big cat). Bürki et al. (2016) further demonstrated that the noun and the determiner (e.g., the cat) are planned sequentially, with the determiner being possibly planned later because it depends on the noun.

The above-mentioned evidence on language planning is purely correlational in nature and hardly any of the studies mentioned provide broad evidence on fronto-temporal areas involved in multi-word production. In comparison to EEG, TMS has a better spatial resolution (1–1.5 cm, Walsh and Rushworth, 1999) and may provide a more fine-grained picture of grammatical processing in the brain. In the current study, we aimed at investigating the causal relevance of intact function in left aIFG and pIFG for the processing of grammatical and lexical information during the production of determiner-adjective-noun phrases. We used Danish stimuli, in which, like in other Germanic languages, the attributive adjective and the determiner agree in gender and number with the noun. Our assumptions about grammatical and lexical items were based on Boye and Harder (2012) usage-based linguistic theory. This theory suggests that the distinction between grammatical and lexical information is based on a semantic distinction pertaining to information prominence: while lexical items can convey the main point (foreground information) of an utterance, grammatical items are conventionalized (hence entrenched) as carriers of secondary (background) information. For instance, in the expression *the boy swims* the words *boy* and *swim* would be lexical items because they can (dependent on context) carry the main point of the utterance. If we omit the grammatical items *the* and *-s*, having *boy swim* left, we could still work out the speaker’s communicative intention. On the contrary, if we omit the lexical items, we would be left with *the*, *-s* and word order, which only provide secondary information and are not sufficient to convey the communicative intention. This implies that these items are structurally different. Since grammatical items are secondary, they are dependent on (and thus require combination with) a host in relation to which they can express their secondary meaning (for example, the definite article *the* in *the cat* cannot be produced in isolation) and they cannot be stressed. A systematic and expected exception to this is found in metalinguistic corrective contexts, such as *I said THE dog, not A*, where conventions may be overridden and grammatical items contrasted in isolation. In contrast, lexical items do not show such a dependency. Accordingly, many lexical items can be the only item in an utterance. This is the case not only with verbs (*Run! Leave! Fire!*), but also with nouns (*Car! Cat! Cab!* uttered as warnings or calls) and adjectives (*Red! Small! Three!* uttered in response to questions regarding color, size, and number, respectively).

Based on this theory, numerals like Danish *en* (“one,” common gender) and *et* (“one,” neuter) are clearly lexical (they can receive focal stress and be produced in isolation), whereas the syntactically similar and nearly homophonous indefinite articles *en* [“a(n),” common gender] and *et* [“a(n),” neuter] are clearly grammatical (they cannot receive focal stress or be produced in isolation, outside of metalinguistic, corrective contexts, where conventions are overridden). Consider, for example, the noun phrase *et rødt brev* (“one/a red letter”). If *et* is read as representing the lexical numeral, it can receive focal stress. If, on the other hand, it is interpreted as representing the indefinite grammatical article, it cannot.

Using a sentence production paradigm, where these closely related numerals and articles are elicited, we expected to find a functional-anatomical double dissociation during language processing, with left aIFG (BA 47) being causally involved in lexical processing and pIFG (BA 44) contributing to grammatical processing (Price, 2010) and more specifically to definite article selection (Heim et al., 2009b). We chose BA 44 and not BA 45 as a target in the left pIFG for grammatical processing to ensure we do not interfere with lexico-semantic processes that BA 45 may be involved with (Hagoort, 2005). This hypothesis was based on two sets of theoretical assumptions (cf. the discussion above): (1) Left aIFG is associated with semantic processing, and lexical items have a heavier semantic load than grammatical items; (2) pIFG is associated with syntactic processing, and unlike lexical items, which can stand alone, grammatical items depend on syntactic processing as they require combination with other items.

We measured reaction times as the primary dependent variable (time from stimulus onset to articulation onset), determiner durations (time from articulation onset to the determiner articulation offset), and adjective-noun phrase durations (time from adjective-noun phrase articulation onset to the phrase articulation offset) as secondary dependent variables. In addition to the grammatical and lexical determiners, the phrase has both grammatical (gender agreement) and lexical (adjectives and nouns) components. Our main hypothesis was that TMS should result in a functional-anatomical double dissociation for both tasks. Specifically, we expected prolonged reaction times in the grammatical task relative to the lexical task with TMS over pIFG. In contrast, TMS over left aIFG should delay response times in the lexical task more than in the grammatical task. As for durations, we predicted that the grammatical determiner duration would be selectively affected by TMS over pIFG and the lexical determiner duration would be selectively affected by TMS over aIFG. It is important to note that, due to differences in stress, the grammatical determiner was significantly shorter than the lexical determiner (Michel Lange et al., 2018). We wish to emphasize that we were interested in the modulatory effects of TMS on the difference in the duration between the grammatical and lexical determiner (i.e., the interaction between TMS site and task), rather than the duration of the grammatical or lexical determiner itself (i.e., the main effect of task). This means that we would expect the difference between lexical and grammatical determiner durations to increase during TMS over pIFG (selective effect on grammatical determiner duration) and to decrease under aIFG stimulation (selective effect on lexical determiner duration).

## Materials and Methods

### Participants

The final sample consisted of 19 healthy native speakers of Danish (8 male, aged between 18 and 34). The data from 11 additional participants were excluded due to technical errors (*n* = 6), unpleasant side effects of the TMS procedure (*n* = 2), including facial twitches and lightheadedness, decision to drop out after the first session (*n* = 1), low accuracy (<80%) in the sham condition (*n* = 1) or invalidly fast response speed (mean response times < 200 ms) (*n* = 1). None of the subjects had a history of neurological or psychiatric disorders and all had (corrected to) normal vision. All participants were screened for MRI and TMS contraindications prior to the experiment (Keel et al., 2001; Shellock and Spinazzi, 2008) and gave written informed consent prior to participation. The study was approved by the local ethics committee.

### Study Design

**FIGURE 1:**
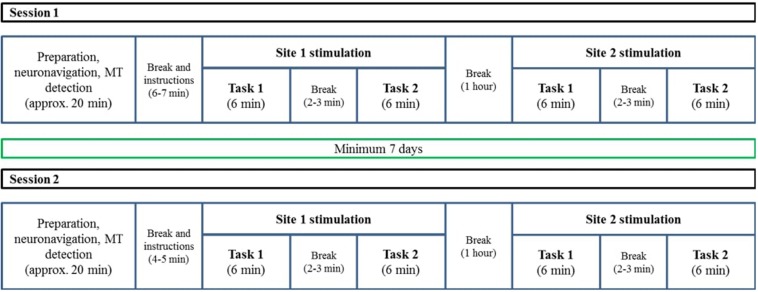
A schematic description of the two TMS sessions. The procedure was similar for both sessions, with the only difference that one of them was a sham session and the other one was an effective stimulation session. The order of TMS condition (sham/effective), tasks (lexical/grammatical), and TMS sites (BA 44/BA 47) were counterbalanced across participants. The T1 acquisition session was conducted on another day and was not part of the TMS sessions.

We used a 2 × 2 × 2 factorial design with the factors TMS site (aIFG vs. pIFG), TMS condition (effective vs. sham), and task (grammatical vs. lexical). Structural MR images for each participant were obtained several days before the experimental sessions. The experiment consisted of two TMS sessions (effective or sham TMS) that were performed with an inter-session interval of at least 1 week to reduce carry-over or learning effects. Both sessions were scheduled on the same day of the week at the same time. Each session started with a preparation stage, where participants received detailed information about the TMS procedure. Thereafter, each participant’s head was co-registered to his or her T1-weighted image with a stereotactic neuronavigation system. Finally, the cortical resting motor threshold (RMT) was determined over the left primary motor hand area. After preparation, experimental instructions were given. Both sessions consisted of two runs, separated by a 1-h break. The runs differed with respect to the TMS site (aIFG vs. pIFG). In each run, the speech-production tasks (lexical and grammatical) were performed in blocks, interspersed with short breaks. The order of sessions, runs and task blocks was counterbalanced across participants (Figure 1).

### Materials and Procedure

We used a paradigm developed by Michel Lange et al. (2017) to test Boye and Harder (2012) theory on the grammar-lexicon distinction. The materials consisted of line drawings of objects that represented the referents of 16 Danish concrete nouns in four colors. Half of the nouns were monosyllabic and the other half were bisyllabic (the full list of the nouns can be found in the Supplementary Material). The experimental paradigm involved a conversation-like setting, where a female voice first presented the picture on the screen (e.g., “I have a/one red letter” for the grammatical task and “I have two red letters” for the lexical condition). Then a blank screen appeared with the same voice asking “What do you have?” (grammatical task) or “How many do you have?” (lexical task). Finally, the target picture appeared with either color contrast (grammatical task) or number contrast (lexical task) and the participant was expected to respond accordingly (*et grønt brev*, which translates as “a green letter” or *et rødt brev* which translates to “one red letter”). In both tasks, the expected responses were phonologically similar and only differed in terms of stress and contrast (number contrast or color contrast). The stress contrast ensured that we were contrasting different items (grammatical vs. lexical), and not different variants of the same item. Additionally, the target phrases in both conditions contained one stressed syllable (the numeral in the lexical condition and the adjective in the grammatical condition).

**FIGURE 2:**
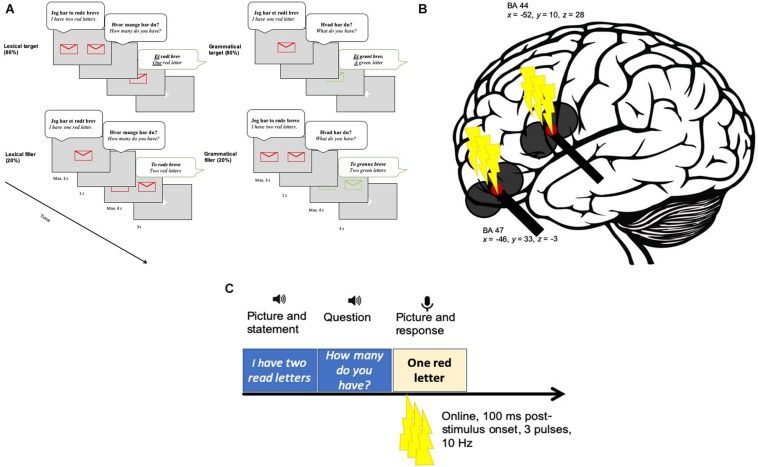
AThe experimental procedure. **(A)** An example of one target (top) and one filler (bottom) trial per lexical (left) and grammatical (right) tasks. Participants saw a picture and simultaneously listened to an auditorily presented stimulus [e.g., *Jeg har to røde breve* (“I have two red letters.”)]. Thereafter, a question was presented via speakers (e.g., *Hvor mange har du?* (“How many do you have?”)]. Next, a picture cued the expected response (e.g., *Et rødt brev* (“One red letter”)]. Eighty percentage of the time the participants were exposed with target items and 20% of the time with filler items. **(B)** The target sites in BA47 (*x* = -46, *y* = 33, *z* = -3) and BA 44 (*x* = -46, *y* = 33, *z* = -3). **(C)** The stimulation in relation to the experimental paradigm. Three TMS pulses were applied 100 ms after target picture onset. A fixation cross appeared before the next trial started.

The number contrast entailed one of the two possible options (one or two), whereas the color contrast entailed one of the four possible options (red, green, blue or yellow). In the color contrast (grammatical) condition each color could be contrasted to any of the other three colors with an equal probability. Twenty percentage of the trials were filler sentences where the expected response involved the numeral two (e.g., “two red letters”) (Figure 2). The filler responses followed either a singular prompt (“I have one red letter. How many do you have?”) or a plural prompt (“I have two red letters. What do you have?”). Thus, in the lexical condition the target response was always “one” and the filler response was always “two.” Similarly, in the grammatical condition the target response was always “a/an” and the filler response was always “two.” The tasks were presented in blocks. Each block consisted of 32 target and 8 filler trials. The target and filler trials were randomized. The experimental paradigm was programmed and presented using PsychoPy v. 1.83.04 (Pierce, 2007) on a screen placed on a 70 cm distance from the participants.

Prior to each experimental session, participants were familiarized with the procedure. To avoid errors due to naming variability, participants were presented with the 16 possible nouns included in the experiment. Accordingly, they first saw object-noun combinations and then had to perform an object naming task without reading the respective noun. Afterward, they practiced several trials of the actual task, and the experimenter corrected them if necessary. Once they had learned the task, the first experimental block began. Responses were recorded using a microphone. Reaction times and durations were extracted with Praat (Boersma and Weenink, 2002) by a phonetically trained assessor naïve to the aims of the experiment. Three different response measures were assessed as dependent variables for the TMS effect. Reaction times (RTs) were measured from stimulus presentation to articulation (as primary dependent variable), whereas duration 1 (Dur1) reflects the length of the production of the grammatical or lexical determiner and duration 2 (Dur2) the length of adjective plus noun (as secondary dependent variables).

### Transcranial Magnetic Stimulation

Each TMS session started with the functional determination of the individual RMT defined as the minimum TMS intensity required to induce a motor-evoked potential in the right abductor pollicis brevis. We used the TMS *Motor Assessment Tool (MTAT 2.0)*, which provides parameter estimation by sequential testing (PEST) procedures using the Maximum-Likelihood strategy for estimating motor thresholds. For the sham condition, 30% of RMT was applied (corresponding to 16–28% of maximal stimulator output) to the same target areas. We chose this form of sham stimulation, as it mimics the effective stimulation as accurately as possible without causing functional effects in the cortex (Sun et al., 2012). For effective stimulation, an intensity of 110% of the individual RMT was used (corresponding to 55–99% of maximal stimulator output).

In the main experiment, a coil holder designed for the TMS robot was attached to the TMS robot arm (*TMS Navigator robotic edition*, Localite GmbH, Sankt Augustin, Germany; Axilum Robotics TMS Robot). The TMS robot has the advantage of increased precision in coil placement and maintenance of the correct position. During each trial, three pulses were applied at a frequency of 10 Hz with the first pulse given 100 ms after presentation onset of the target picture (see Figure 2). TMS was given 100 ms post-onset because we did not want to perturb early visual processing. A similar timing was used in previous TMS studies targeting the left IFG during different language comprehension tasks (e.g., Devlin et al., 2003; Gough et al., 2005; Hartwigsen et al., 2010a, b, 2015). Based on these previous studies, we believe that our 300 ms burst should have interfered with processing in a task-relevant time window.

In all conditions, biphasic TMS pulses were applied using a focal *MagVenture Small Cool-Butterfly B35* coil (inner diameter 23 mm, outer diameter 46 mm) connected to a *MagVenture X100* stimulator (MagVenture A/S, Farum, Denmark). Note that previous studies have shown a functional-anatomical double dissociation for phonological vs. semantic aspects of language comprehension within the left IFG using even larger TMS coils (e.g., Gough et al., 2005; Hartwigsen et al., 2010b). Therefore, in the current study the use of a smaller butterfly coil ensures even higher focality (Deng et al., 2013). The overall application of TMS pulses was within the recommended safety guidelines (Rossi et al., 2009; Rossini et al., 2015).

### Localization of TMS Target

The IFG sites were targeted using a robotized TMS system (*TMS Navigator robotic edition*, Localite GmbH, Sankt Augustin, Germany; Axilum Robotics TMS Robot). As a prerequisite for stereotactical neuronavigation, structural MR images were obtained prior to the TMS session at a *Philips Achieva 3-Tesla* scanner (Philips Achieva, Best, The Netherlands; sequence parameters: 190 slices, TE = 0.000 ms, TR = 5.98 s, 1 mm isotropic resolution). The participants’ heads were co-registered with the individual T1-weighted image. Mean Talairach coordinates for the target sites were obtained from previous neuroimaging studies [aIFG (BA 47): *x* = -46, *y* = 33, *z* = -3; Zhu et al., 2015; pIFG (BA 44): *x* = -52, *y* = 10, *z* = 28; Dapretto and Bookheimer, 1999] and converted to MNI coordinates. The respective individual target sites were determined in the neuronavigation software and the robot arm moved to the target area accordingly. The above-mentioned procedure allows precise targeting of the coordinates of interest (cf. Hartwigsen et al., 2010b).

### Data Analysis

A response was counted as correct if all the components of the expected response (determiner, adjective and noun) were present, the agreement was grammatically correct, and the noun and the color adjective were identical to those of the expected response. Incorrect responses (2.5% of all trials) were excluded from data analyses. We also removed outliers more than three standard deviations away from the mean (42 observations in total). As the accuracy was high, we did not analyze error rates.

Separate linear mixed models were computed to analyze RT, Dur1, and Dur2 data. The TMS site (pIFG vs. aIFG), TMS condition (effective vs. sham), and task (grammatical vs. lexical) were defined as fixed effect variables. Participants and items were included in the models as random effects. Data were analyzed using the lme4 package (Bates et al., 2015) in R (R Core Team, 2014). We first built a complex model with TMS site, TMS condition and task as main effects, their interactions as fixed effect variables and participants and items as random intercepts and slopes. To allow convergence, we set the number of iterations to 100,000 and “bobyqa” optimizer. If the model did not converge, we removed random slopes one by one until it converged. A possible cause of lack of convergence was overparameterization (Bates et al., 2015).

Then the step function from the same package was used to determine the best fitting model through backwards reduction. We further plotted the residuals to check for normality. For all the dependent variables the right tail of the distribution was not well-estimated by the model. We thus log-transformed the dependent variables and repeated the same procedure. Additionally, to further test our hypotheses we obtained estimated marginal means, using the emmeans and contrast functions in the emmeans package with multivariate *t*-distribution (mvt) adjustment to control for multiple comparisons (Lenth, 2019).

The filler item RTs were analyzed separately, to ensure that the observed effects were due to differences in grammatical and lexical determiner retrieval and not processes involved in color and numeral retrieval. We followed the exact same procedure for fillers as for target items, removing outliers three standard deviations above the mean, log transforming the data and building a model. For exploratory purposes, we also built linear models to investigate the relationship between ΔRT (RT_*sham*_ - RT_*effective*_) and ΔDur1 (Dur1_*sham*_ – Dur1_*effective*_) and between ΔRT and ΔDur2 (Dur2_*sham*_ - Dur2_*effective*_). To this end, we used the mean values for each participant per task and site for all the dependent variables, leading to four data points per participant.

## Results

**FIGURE 3:**
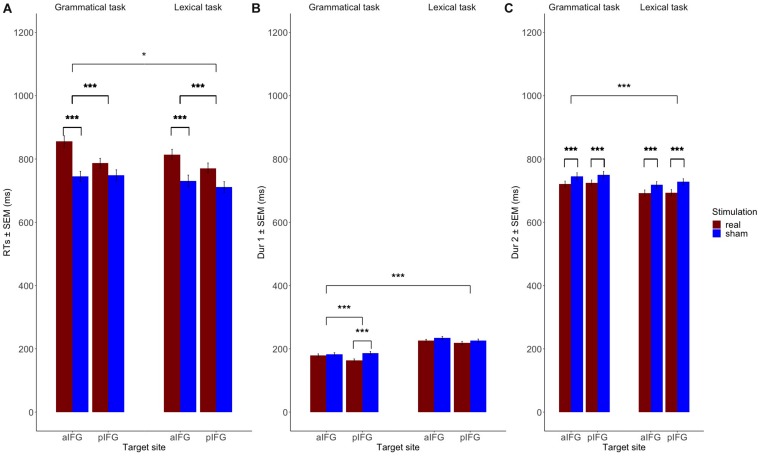
Mean group data (*n* = 19). Fitting the data to linear mixed models showed that **(A)** Response times (RTs) are significantly increased both for the grammatical and the lexical task over both aIFG and pIFG in the effective TMS condition compared to sham. RT prolongation is relatively stronger in the grammatical task than the lexical task when TMS targets aIFG compared to TMS over pIFG (TMS condition by TMS site by task 3-way interaction). **(B)** The grammatical determiner duration (Dur1) is selectively decreased when effective TMS targets pIFG (TMS condition by TMS site by task 3-way interaction). **(C)** The adjective-noun phrase duration (Dur2) is affected by effective stimulation, independent of the stimulation site. Dur2 is generally shorter in the lexical task than the grammatical one due to stress on the adjective-noun phrase on the latter.

The mean response times and durations for all conditions are given in Table 1 and illustrated in Figure 3. The statistical results are summarized in Table 2.

**TABLE 1:** Mean ± SE reaction times (RT), Dur1, and Dur2 for TMS site (pIFG and aIFG), TMS condition (effective and sham), and task (grammatical and lexical).

	**Effective TMS**				**Sham TMS**	
***n* = 19**	**RT ± SE (ms)**	**Dur1 ± SE (ms)**	**Dur2 ± SE (ms)**	**RT ± SE (ms)**	**Dur1 ± SE (ms)**	**Dur2 ± SE (ms)**
**pIFG**						
Grammatical	787 ± 9	163 ± 2	724 ± 5	750 ± 8	186 ± 3	750 ± 5
Lexical	770 ± 9	218 ± 3	694 ± 5	721 ± 10	226 ± 2	728 ± 5
**aIFG**						
Grammatical	856 ± 9	178 ± 2	721 ± 5	749 ± 9	183 ± 3	745 ± 6
Lexical	815 ± 9	225 ± 2	693 ± 5	741 ± 10	234 ± 2	5

**TABLE 2:** Summary of the logRT, logDur1, and logDur2 results.

	**logRT**				**logDur1**				**logDur2**			
	***b***	***SE***	***t***	***p***	***b***	***SE***	***t***	***p***	***b***	***SE***	***t***	***p***
Intercept	6.64	0.05	128	< 0.001	5.04	0.05	100	< 0.001	6.6	0.03	181	< 0.001
TMS condition	–0.06	0.01	–5.6	< 0.001	0.12	0.01	6.8	< 0.001	0.04	0.03	13.6	< 0.001
Task	–0.03	0.01	–2.6	0.016	0.31	0.01	24.3	< 0.001	–0.04	0.03	–13.1	< 0.001
Site	0.08	0.01	7.4	< 0.001	0.08	0.01	6.8	< 0.001	0.04	0.03	–3.1	0.002
TMS condition × Task	–0.04	0.02	–2.4	0.02	–0.09	0.02	–4.9	< 0.001				
TMS condition × TMS site	–0.8	0.02	–5.6	< 0.001	–0.1	0.02	–5.5	< 0.001				
TMS site × Task	–0.02	0.02	–1.2	0.21	–0.05	0.02	–2.8	0.005				
TMS condition × Task × TMS site	0.05	0.02	2.1	0.03	17	5	3.5	< 0.001				

*Site (aIFG and pIFG, where pIFG is the reference), Task (grammatical and lexical, where grammatical is the reference) and Stimulation (effective and sham, where effective is the reference).*

### Response Times (RTs)

Random slopes were removed from the model one at a time until the model converged, which left it with the three fixed effects and their three-way interaction and participant and item as random intercepts. The step function through backwards reduction suggested a best fitting model that included the three-way interaction of the fixed effects [*F*(4629) = 4.8, *p* = 0.029]:

l⁢o⁢g⁢R⁢T∼T⁢M⁢S⁢c⁢o⁢n⁢d⁢i⁢t⁢i⁢o⁢n*T⁢M⁢S⁢S⁢i⁢t⁢e*T⁢a⁢s⁢k+(1|p⁢a⁢r⁢t⁢i⁢c⁢i⁢p⁢a⁢n⁢t)

 +(1|i⁢t⁢e⁢m)

There was a significant three-way interaction between TMS condition, TMS site and task [*b* = 0.05 ± 0.02, *t*(4629) = 2.2, *p* = 0.03], indicating that effective TMS differentially affected both tasks depending on the site of stimulation. Inspection of the group data showed that the interaction was caused by a relative delay in mean logRT during effective TMS trials relative to sham TMS trials [main effect of TMS condition: [*b* = -0.06 ± 0.01, *t*(4629) = -5.6, *p* < 0.001], with the delays being stronger when aIFG was stimulated, compared to pIFG (TMS condition by TMS site interaction: [*b* = -0.08 ± 0.02, *t*(4629) = -5.6, *p* < 0.001] (Figure 3A). This effect was relatively stronger during the grammatical task compared to the lexical task (as reflected in the 3-way interaction of TMS condition, TMS site and task, see above) (Figure 3A and Table 3). *Post-hoc* testing revealed that mean logRTs were more prolonged by effective TMS of aIFG in the grammatical task than in the lexical task [ΔlogRT = 0.05 ± 0.01, *t*(4630) = 4.1, *p* < 0.001]. There was no evidence for logRTs being differentially affected by effective TMS over pIFG in the grammatical task relative to the lexical task [ΔlogRT = -0.03 ± 0.01, *t*(4630) = -2.4, *p* = 0.15].

**TABLE 3:** Estimated marginal mean contrasts for logRTs for TMS condition (effective vs. sham), TMS site (pIFG vs. aIFG), and task (grammatical vs. lexical), using mvt adjustment.

**Contrast**	**Δ logRT**	***SE***	***df***	***t***	***p***
Effective, gram, pIFG – sham, gram, pIFG	0.03	0.01	4630	2.4	0.147
Effective, gram, pIFG – effective, lex, pIFG	0.03	0.01	4630	4.1	< 0.001
Effective, gram, pIFG – effective, gram, aIFG	0.08	0.01	4633	7.4	< 0.001
Effective, lex, pIFG – sham, lex, pIFG	0.10	0.01	4629	8.9	< 0.001
Effective, lex, pIFG – effective, lex, aIFG	0.06	0.06	4630	5.0	< 0.001
Effective, gram, aIFG – sham, gram, aIFG	0.14	0.01	4630	13.4	< 0.001
Effective, gram, aIFG – effective, lex, aIFG	0.05	0.01	4630	8.9	< 0.001
Effective, lex, aIFG – sham, lex, aIFG	0.13	0.01	4630	12.4	< 0.001

As for filler logRT model, in addition to the random slopes, item had also to be removed because of singularity. Model analysis showed no significant 3-way interaction [*b* = -0.03 ± 0.04, *t*(1131) = ±0.7, *p* = 0.47]. There was, however, a TMS site by TMS condition interaction (*b* = -0.07 ± 0.04, *t*(1131) = -2.5, *p* = 0.01). This interaction was a result of a stronger TMS-induced delay for aIFG relative to pIFG [main effect of TMS site: *b* = 0.7 ± 0.02, *t*(1131) = 3.1, *p* = 0.02]. Thus, the delay in logRTs for the filler items was comparable to those of the target items, except for the differential effect on the grammatical task compared to the lexical task for TMS over aIFG. More details about the filler model can be found in the Supplementary Material.

### Durations

The best fitting model for *Dur1* included stimulation, task and site as fixed effect variables, their three-way interaction, and participant and item as a random effect variables [*F*(4629) = 15, *p* < 0.001]. Random slopes were removed from the model due to convergence issues.

l⁢o⁢g⁢D⁢u⁢r⁢1∼T⁢M⁢S⁢C⁢o⁢n⁢d⁢i⁢t⁢i⁢o⁢n*T⁢M⁢S⁢S⁢i⁢t⁢e*T⁢a⁢s⁢k+(1|p⁢a⁢r⁢t⁢i⁢c⁢i⁢p⁢a⁢n⁢t)+(1|i⁢t⁢e⁢m)

The mixed effects model yielded a three-way interaction for Dur1 [TMS stimulation × TMS site × task, *b* = 0.1 ± 0.03, *t*(4629) = 3.9, *p* < 0.001]. Inspection of the group data showed that Dur1 was consistently shorter for grammatical than for lexical determiners across all TMS conditions of TMS, also reflected by a main effect of task (Figure 3B and Table 4). *Post-hoc* analyses revealed that the three-way interaction was driven by a selective TMS-induced (i.e., effective vs. sham) decrease in logDur1 for grammatical determiners when effective TMS was given over pIFG [ΔlogDur1 = -0.12 ± 0.03, *t*(4629) = -9.5, *p* < 0.001]. LogDur1 of the lexical determiners was not shortened by effective TMS over pIFG [ΔlogDur1 = -0.03 ± 0.03, *t*(4629) = -2.4, *p* = 0.14]. Grammatical determiners were not affected by effective TMS of aIFG [ΔlogDur1 = -0.02 ± 0.02, *t*(4629) = -1.7, *p* = 0.53] and neither were lexical determiners [ΔlogDur1 = -0.03 ± 0.03, *t*(4629) = -2.5, *p* = 0.1]. Consequently, we infer that the three-way interaction between TMS condition, TMS site and task was driven by a pre-existing difference between the grammatical and lexical determiners. However, this difference selectively increased under effective TMS targeting pIFG (Figure 3B).

**TABLE 4:** Estimated marginal mean contrasts for logDur1 for TMS condition (effective vs. sham), TMS site (aIFG and pIFG), and task (grammatical vs. lexical), using mvt adjustment.

**Contrast**	**Δ logDur1**	***SE***	***df***	***t***	***p***
Effective, gram, pIFG – sham, gram, pIFG	–0.12	0.01	4629	–9.4	< 0.001
Effective, gram, pIFG – effective, lex, pIFG	–0.32	0.01	4629	–24.3	< 0.001
Effective, gram, pIFG – effective, gram, aIFG	–0.09	0.01	4629	–6.8	< 0.001
Effective, lex, pIFG – sham, lex, pIFG	–0.03	0.01	4629	–2.4	0.14
Effective, lex, pIFG – effective, lex, aIFG	–0.04	0.01	4629	–2.8	0.049
Effective, gram, aIFG – sham, gram, aIFG	–0.02	0.01	4629	–1.7	0.53
Effective, gram, aIFG – effective, lex, aIFG	–0.26	0.01	4629	–20.3	< 0.001
Effective, lex, aIFG – sham, lex, aIFG	–0.03	0.01	4629	–2.5	0.1

For logDur2, the step function fitted a model with TMS condition [*F*(4607) = 186, *p* < 0.001], TMS site [*F*(4607) = 8.1, *p* = 0.004], and task [*F*(4607) = 173.5, *p* < 0.001] as main effect fixed effect variables and participant and item as random effect variables.

l⁢o⁢g⁢D⁢u⁢r⁢2∼T⁢M⁢S⁢c⁢o⁢n⁢d⁢i⁢t⁢i⁢o⁢n+T⁢M⁢S⁢s⁢i⁢t⁢e+t⁢a⁢s⁢k+(1|p⁢a⁢r⁢t⁢i⁢c⁢i⁢p⁢a⁢n⁢t)+(1|i⁢t⁢e⁢m)

Overall, the log duration of the adjective-noun phrase was shorter for effective relative to sham TMS [*b* = 0.04 ± 0.003, *t*(4607) = 13.6, *p* < 0.001]. Similarly, the overall log duration of the phrase was shorter in the lexical task compared to the grammatical one [*b* = -0.04 ± 0.003, *t*(4607) = -13.1, *p* < 0.001]. There was also main effect of site with log duration being higher over pIFG than aIFG in effective stimulation [*b* = -0.1 ± 0.003, *t*(4607) = −3.1, *p* = 0.002]. *Post-hoc* pairwise comparisons, however, did not confirm the TMS site effect for effective stimulation [ΔlogDur2 = -0.005 ± 0.01, *t* = -1.3, *p* = 0.78 for the lexical task and ΔlogDur2 = -0.01 ± 0.01, *t* = -1.2, *p* = 0.82]. The summary of the RT, Dur1, and Dur2 linear mixed model results can be found in Table 2.

### Correlation Between Response Times and Durations

**FIGURE 4:**
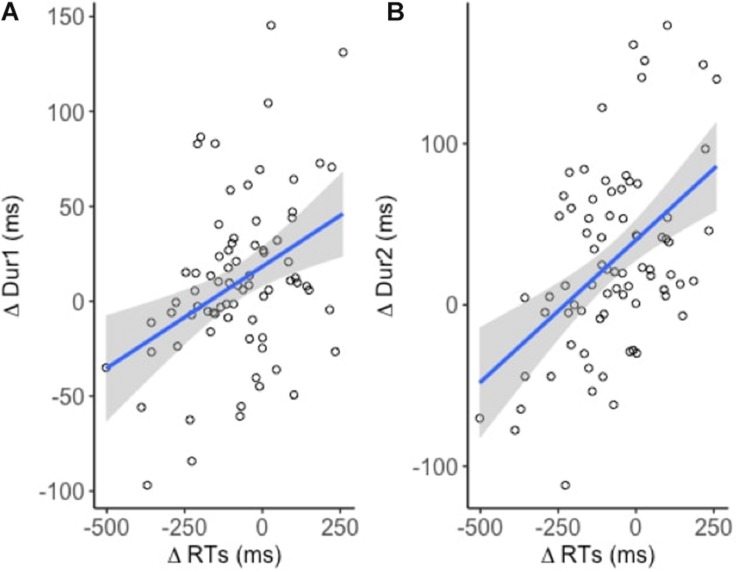
Linear models fitted to the dependent variables (*n* = 19). Significant positive relationship between **(A)** ΔRT and ΔDur1 and **(B)** ΔRT and ΔDur2.

A weak positive correlation between ΔRT and ΔDur1 indicated that increased reaction times lead to increased response durations [*R*^2^ = 0.14; *t*(74) = 3.5, *p* < 0.001] (Figure 4A). A similar but relatively stronger relationship was observed between ΔRT and ΔDur [*R*^2^ = 0.23, *t*(74) = 4.7, *p* < 0.001] (Figure 4B). However, neither of the effects were task or site specific.

## Discussion

To the best of our knowledge, the present study represents the first investigation of the causal role of different IFG subregions in lexical and grammatical processing underpinning multi-word utterances. Employing a well-matched language production paradigm, we applied focal TMS to anterior or posterior left IFG, when healthy participants had to produce a multi-word utterance starting with a lexical or grammatical determiner. Focal TMS evoked two site-specific and determiner-specific functional lesion effects: First, TMS induced a delay in response times that was more pronounced in the grammatical condition compared to the lexical condition, when TMS targeted the aIFG (BA 47). The stronger TMS effect on the grammatical task was unexpected, since we had predicted a stronger interference with the lexical determiner condition, when targeting a key semantic area. Nevertheless, this effect indicates a specificity of the TMS-induced disruption. Second, TMS over left pIFG (BA 44) selectively shortened articulation time for grammatical determiners relative to lexical determiners. Together, these two TMS-induced changes in task performance indicate a joint involvement of the anterior and posterior part of left IFG in the implementation of grammatical determiners during language production, suggesting an involvement of aIFG in the initiation and pIFG in the production of grammatically appropriate responses at the sentence level.

Together, these results suggest that both the anterior and posterior part of the IFG contribute to grammar processing without providing strong evidence for the previously reported division of labor in the left IFG, with a key role of pIFG in grammar and phonological processing and a stronger contribution of aIFG in semantic processing (Caplan et al., 1998; Dapretto and Bookheimer, 1999; Sakai et al., 2002; Devlin et al., 2003; Hagoort et al., 2004; Gough et al., 2005; Friederici, 2009; Hartwigsen et al., 2016; Kuhnke et al., 2017). The apparent discrepancy between our study and previous work might be attributed to differences in the nature of the experimental task. While the above-cited previous studies employed language comprehension tasks, we used a similarly complex noun-phrase processing paradigm during language production. Therefore, it is conceivable that the functional contributions of aIFG and pIFG observed in our study only emerge in the context of overt language production.

The site-specific decrease in determiner duration during TMS of pIFG observed for articulation duration in the grammatical determiner condition might be interpreted as evidence for the involvement of pIFG in the phonological aspects of grammatical item retrieval. This would imply a processing overlap for grammar and phonology at the exact same area. This notion is supported by previous neuroimaging and TMS studies which have demonstrated the involvement of the left pIFG in syntactic and phonological processes (Sakai et al., 2002; Nixon et al., 2004; Gough et al., 2005; Hartwigsen et al., 2010a, b; Acheson and Hagoort, 2013). Initially, we did not expect to modulate phonological processes in our study. We carefully chose a target site that has been shown to be engaged in grammatical processes (Dapretto and Bookheimer, 1999) and a stimulation time window 100 ms post-stimulus to avoid interference with phonological planning, as the phonological word retrieval takes place around 355 ms post-stimulus (Indefrey and Levelt, 2004). Notably, the information on the precise timing of different processes during language production is mainly derived from studies using picture naming which might substantially differ from the task under investigation in our study. On the other hand, it should be borne in mind that a number of previous TMS studies associated the left pIFG with phonological processing and disruption of this area was demonstrated to significantly delay phonological response speed during language comprehension tasks, when TMS was given at a time window between 100 and 400 ms after word onset during the presentation of either visual or auditory stimuli (e.g., Gough et al., 2005; Romero et al., 2006; Hartwigsen et al., 2010a, b; Hartwigsen et al., 2015). In a recent EEG study, Bürki et al. (2016) have shown that phonological retrieval for determiners in a determiner-adjective-noun phrase is at 300 ms post-stimulus and that it precedes the gender agreement phase. It is thus possible that in our study we interfered with the phonological component of grammatical item retrieval and therefore we did not observe differences in response times over pIFG between the grammatical and the lexical tasks, but a selective acceleratory effect on articulation duration for the grammatical determiner condition, impacting on the phonological component.

pIFG has also been associated with the stress in speech production (Peschke et al., 2012). Given that our target items differed in stress, one could argue that the observed selective decrease in grammatical determiner duration was due the fact that the grammatical determiner was unstressed, whereas the lexical determiner was stressed. However, we did not observe a selective decrease in the unstressed adjective-noun phrase duration in the lexical condition, suggesting that our findings in determiner duration decrease are not entirely due to the fact that the grammatical item was unstressed.

One possible explanation for the observed decrease in response duration for the grammatical determiner is that TMS facilitated phonological planning during language production. The observed decrease in the determiner duration for the grammatical task might also indicate that our TMS protocol primed activity in the stimulated left pIFG to a level that was optimal for task processing and thus facilitated phonological planning. Indeed, it was previously argued that the effects of TMS on neural activity might not necessarily disrupt behavior, but it may also give rise to a “paradoxical improvement” in task performance (Pascual-Leone et al., 2000; Hartwigsen et al., 2015). Several studies reported faster response speed with different TMS protocols over a language area (Sparing et al., 2001; Nixon et al., 2004; Andoh et al., 2006; Sliwinska et al., 2017). Consequently, one may assume that when TMS is applied to a region that is expected to be involved in a given task *before* the cognitive process is executed, the initial neuronal activation state of that region is altered (i.e., suppressed), causing divergent behavioral effects (Stoeckel et al., 2009; Sandrini et al., 2011). In line with this argumentation, a previous study that also employed a 10 Hz TMS protocol during language production reported facilitation of phonological response speed in a rhyme generation task when TMS was given early after stimulus onset (Klaus and Hartwigsen, 2019). In that study, it was argued that TMS might have rather increased the amount of activity in the targeted pIFG to a level that was optimal for task performance, potentially resulting in a “preactivation” of phonological activity (see Töpper et al., 1998; Sparing et al., 2001, for a similar reasoning). This explanation is supported by a number of previous studies that reported behavioral facilitation when TMS was given immediately before picture naming over left-hemispheric language areas (Töpper et al., 1998; Mottaghy et al., 1999; Wassermann et al., 1999; Sparing et al., 2001). Consequently, the observed decrease of the determiner duration for the grammatical task in our study may have resulted from phonological priming. Of note, earlier studies reported reduced response times (initiation), whereas we found a reduction in duration for articulation (execution), suggesting that paradoxical improvement may extend to language production. Moreover, despite the observed difference at the execution level (i.e., decreased duration), it is possible that the facilitation occurred already at the initiation phase when the phonological planning of the grammatical item retrieval occurred, as articulation takes place at an even later point after stimulus onset and involves more posterior cortical regions (Indefrey, 2011).

Another main finding was that effective TMS over aIFG significantly delayed the initiation of a verbal response in both tasks, but the TMS-induced delay was significantly stronger in the grammatical condition. While the latter finding was somehow unexpected, it may be explained in terms of aIFG being more involved in semantic processes. According to Boye and Harder (2012), there are semantic differences between the grammatical determiner (background information) and the lexical numeral (foreground information), and the lexical numeral should have more semantic weight. It is possible that planning of background information is more demanding and hence the grammatical determiner retrieval has more semantic weight, rendering this experimental condition sensitive to the disruptive effects of TMS targeting aIFG.

The difference in the outcome of the previous TMS studies on language comprehension mentioned above and our present study might be explained by several differences in the employed design. In contrast to the previous studies, we used a multi-word production paradigm, which arguably mirrors natural language production more closely than single-word paradigms. Moreover, our two experimental tasks were well-matched and differed only in terms of stress. Thus, both the grammatical and the lexical outputs had grammatical (e.g., agreement) and lexical (e.g., adjective) components and only the determiner or the numeral weighed in one direction or the other. This may be the reason why the response times were delayed for both tasks by TMS over both aIFG and pIFG. The absence of a task-specific effect for TMS over pIFG might indicate that potential differences were too subtle to be measured in response times. Moreover, we also observed a weak relationship between ΔDur1 and ΔRT that was neither task- nor site-specific. This finding may indicate that the TMS-induced response time delay was compensated by a decrease in determiner or numeral duration and therefore a subtle effect could not be observed.

The lack of any task- and site-specific effects on the adjective-noun duration shows that our paradigm was well-matched. The number of grammatical and lexical items in the adjective-noun phrase across grammatical and lexical tasks was similar, and the only difference was in the article (grammatical task) or the numeral (lexical task) preceding the adjective-noun phrase. This paradigm allowed us to investigate differences in the processing of the target grammatical and lexical determiners without too much interference from the adjectives and nouns. The non-specific decrease in adjective-noun duration for effective TMS relative to sham TMS is an indicator of a general TMS effect on adjective-noun phrase duration. We wish to emphasize that additional analysis revealed a significant correlation between delayed response times and decreased durations, which likely indicates that the delayed response times were compensated by faster durations. This mechanism might represent a general TMS effect that was independent of the specific task. Another possible alternative explanation is related to the time point of the interference. Our TMS burst was applied 100–300 ms after stimulus onset and might not have interfered with the planning of the adjective and noun. To further address this issue, chronometric TMS studies may disentangle the temporal contribution of left aIFG and pIFG in multi-word production.

A potential limitation, however, is due to the slight difference in task demands in terms of the grammatical and the lexical conditions. Namely, in the grammatical task the participants had to retrieve one of the four possible colors in every trial, as the color of the cue and the expected response did not match (color contrast). In the lexical task, they had to retrieve one of the two numerals instead (number contrast) and the color of the expected response was similar to the color of the cue. Therefore, the grammatical RTs could have been delayed due to the semantic weight of color and not determiner retrieval. We believe that our additional analysis of the filler items, however, should control for this potential issue. In the filler items, instead of the determiners et/en, the numeral to (“two”) was produced in both the grammatical and lexical task but otherwise the task demands remained the same with color contrast in the grammatical task and number contrast in the lexical task. Thus, if our findings were due to color retrieval, we would expect a similar three-way interaction (TMS condition × TMS site × task) for the filler items as well. However, the filler analysis showed no dissociation in RTs for aIFG stimulation.

We wish to emphasize that we took great care to ensure precise stimulation of the target areas (i.e., by using a small and focal coil, employing co-registration of the participants’ head to their individual T1-weighted images with a stereotactic neuronavigation system and a robot arm holding the coil). Yet, we cannot rule out that BA 45, which is anatomically located between BA 47 and BA 44, was co-stimulated when targeting either BA 47 or BA 44. Given the functional gradient in the left IFG suggested by Hagoort (2005), our findings may not strictly argue for a difference between BA 47 and BA 44, but rather favor an anterior to posterior gradient of grammatical processing in the left IFG.

Despite the challenges of a multi-word production paradigm in a TMS study and the partly unexpected effects, we succeeded in showing a functional involvement of left IFG in grammatical processing during language production. Specifically, pIFG may be involved in the phonological aspects of grammatical processing, whereas aIFG may be responsible for processing semantic information of grammatical and lexical items. Using the same behavioral paradigm, in both healthy subjects and agrammatic patients, our group found converging evidence that grammatical items are processed differently from lexical items (Ishkhanyan et al., 2017, 2019; Michel Lange et al., 2017, 2018; Boye and Bastiaanse, 2018; Nielsen et al., 2019). Together, the results encourage future research into the joint contributions of frontal and parietotemporal brain regions to these processes during language production.

## Data Availability Statement

All datasets generated for this study are included in the article/Supplementary Material.

## Ethics Statement

The studies involving human participants were reviewed and approved by Videnskabsetisk Komité, Region H, Denmark (approval 52703, journal number H-16021767, approved on 14 June 2016). The patients/participants provided their written informed consent to participate in this study.

## Author Contributions

BI has contributed to the design, setting up and programming the experiment, collecting the data, analyzing the data, writing the manuscript, and revisions. VM has contributed to the behavioral aspects of the design, programming the experiment, data collection, and writing the manuscript. KB has contributed to the theoretical aspects of the design, interpretation of the results, and revisions of the manuscript. JM has contributed to the design and the revisions of the manuscript. AK has contributed to the technical aspects of transcranial magnetic stimulation, writing, and revising the manuscript. GH and HS have supervised the whole process of designing, programming and running the experiment, data analysis, interpretation of the results, and writing the final version of the manuscript.

## Conflict of Interest

HS has received honoraria as speaker from Sanofi Genzyme, Denmark and Novartis, Denmark, as consultant from Sanofi Genzyme, Denmark and as senior editor (NeuroImage) from Elsevier Publishers, Amsterdam, Netherlands. HS has received royalties as book editor from Springer Publishers, Stuttgart, Germany. The remaining authors declare that the research was conducted in the absence of any commercial or financial relationships that could be construed as a potential conflict of interest.
