# Supplementary material for: Anterior and Posterior Left Inferior Frontal Gyrus Contribute to the Implementation of Grammatical Determiners During Language Production
Source: Front Psychol. 2020 Apr 27;11:685. doi: 10.3389/fpsyg.2020.00685 (PMC7197372; doi:10.3389/fpsyg.2020.00685)

TMS

Anonymyzed

6/13/2019

```
library(tidyverse)
```

```
## -- Attaching packages --------------------------------------------------------------------- tidyverse

## v ggplot2 3.2.1     v purrr   0.3.3
## v tibble  2.1.3     v dplyr   0.8.3
## v tidyr   1.0.0     v stringr 1.4.0
## v readr   1.3.1     v forcats 0.4.0

## -- Conflicts ---------------------------------------------------------------------- tidyverse_confl
## x dplyr::filter() masks stats::filter()
## x dplyr::lag()    masks stats::lag()
```

```
library(MASS)
```

```
##
## Attaching package: 'MASS'

## The following object is masked from 'package:dplyr':
##
##     select
```

```
library(ggplot2)
setwd("~/Dropbox/TMS frontiers/to submit/resubmission/Frontiers resubmission after review/")

# loading the dataset
data=read.csv("tms.csv", header=TRUE)
d = read.csv("tms_messy.csv")
data$session = d$session_day
write.csv(data, "tms_session.csv")

# keeping the filler with target trials, excluding incorrect responses and outliers


# Excluding problematic participants
data =data[!data$participant=="X89835" & !data$participant=="X903298" & !data$participant=="X02940" & !

# converting seconds to ms
data$rt=1000*data$rt
data$dur1=1000*data$dur1
data$dur2=1000*data$dur2

# Checking the RTs for session 1 and 2
summary(subset(data, session == 1)$rt)
```

```
##    Min. 1st Qu.  Median    Mean 3rd Qu.    Max.
##   222.0   651.0   792.0   821.1   957.0  2880.0
```

```
summary(subset(data, session == 2)$rt)
```

```
##    Min. 1st Qu.  Median    Mean 3rd Qu.    Max.    NA's
```

```
##   247.0   594.0   715.0   739.3   861.0  1901.0      12
```

```
data$session = as.factor(data$session)
ggplot(data, aes(x = rt, fill = session)) + geom_density(alpha = 0.2)
```

```
## Warning: Removed 12 rows containing non-finite values (stat_density).
```

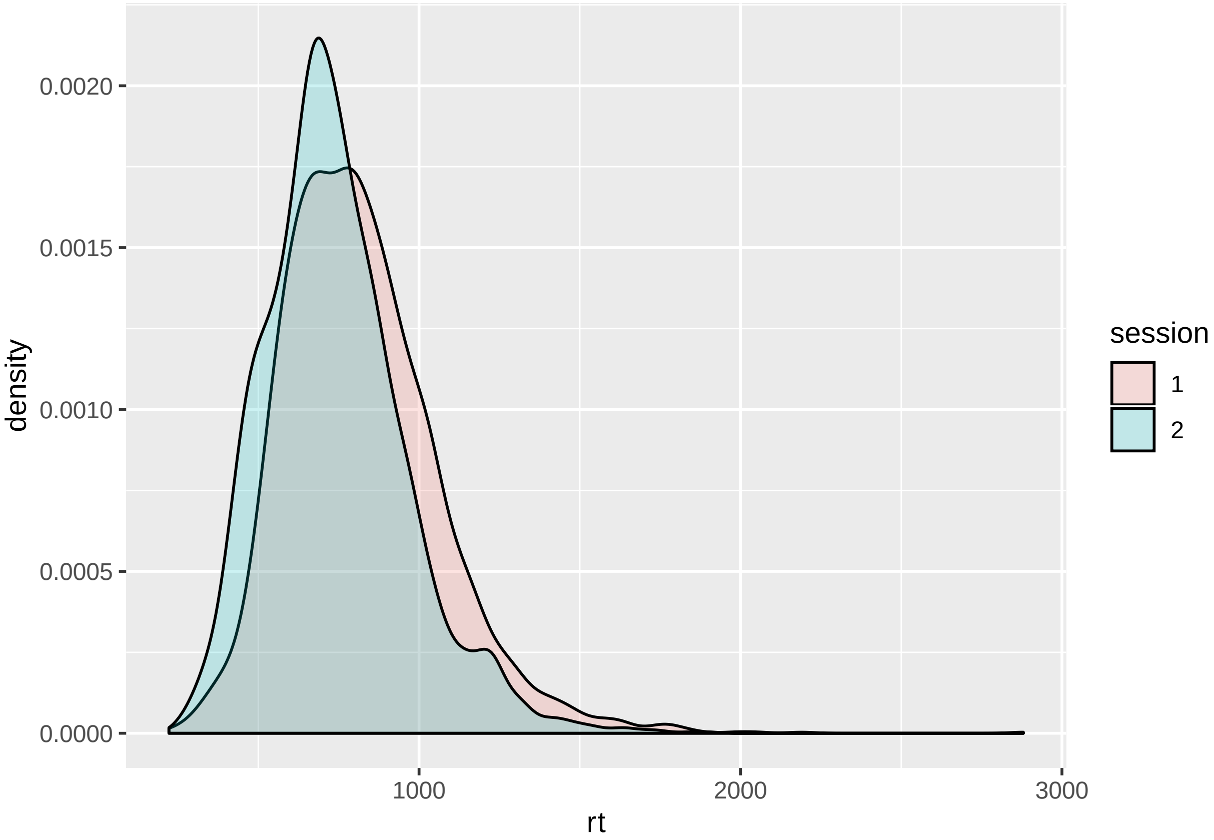


```
# taking only target and correct responses
target=data[which(data$target_filler=="target" & data$accuracy > 0),]


higher_end_rt = mean(target$rt)+3*sd(target$rt) # 42
sum(target$rt > higher_end_rt)
```

```
## [1] 45
```

```
mean(target$rt)
```

```
## [1] 778.5799
```

```
lower_end_rt = mean(target$rt)-3*sd(target$rt)
sum(target$rt < lower_end_rt) # 0
```

```
## [1] 0
```

```
target = target[which(target$rt<higher_end_rt),]
target$logrt = log(target$rt)
```

```
##Descriptive stats
library(pastecs)
```

```
##
## Attaching package: 'pastecs'

## The following objects are masked from 'package:dplyr':
##
##     first, last

## The following object is masked from 'package:tidyr':
##
##     extract
```

```
by(data$rt, list(data$area, data$sham.real, data$gram.lex), stat.desc, basic=FALSE)
```

```
## : BA44
## : real
## : gram
##       median         mean      SE.mean CI.mean.0.95          var      std.dev
## 7.605000e+02 7.950368e+02 7.509307e+00 1.474148e+01 4.285617e+04 2.070173e+02
##     coef.var
## 2.603871e-01
## ------------------------------------------------------------
## : BA47
## : real
## : gram
##       median         mean      SE.mean CI.mean.0.95          var      std.dev
## 8.405000e+02 8.700289e+02 8.406720e+00 1.650319e+01 5.371144e+04 2.317573e+02
##     coef.var
## 2.663788e-01
## ------------------------------------------------------------
## : BA44
## : sham
## : gram
##       median         mean      SE.mean CI.mean.0.95          var      std.dev
## 7.150000e+02 7.616882e+02 8.260781e+00 1.621669e+01 5.186278e+04 2.277340e+02
##     coef.var
## 2.989859e-01
## ------------------------------------------------------------
## : BA47
## : sham
## : gram
##       median         mean      SE.mean CI.mean.0.95          var      std.dev
## 7.420000e+02 7.633276e+02 8.480090e+00 1.664722e+01 5.465306e+04 2.337799e+02
##     coef.var
## 3.062642e-01
## ------------------------------------------------------------
## : BA44
## : real
## : lex
##       median         mean      SE.mean CI.mean.0.95          var      std.dev
## 7.515000e+02 7.616145e+02 7.682279e+00 1.508104e+01 4.485324e+04 2.117858e+02
##     coef.var
## 2.780748e-01
## ------------------------------------------------------------
## : BA47
## : real
## : lex
```

```
##       median         mean      SE.mean CI.mean.0.95          var      std.dev
## 7.830000e+02 8.172527e+02 8.033645e+00 1.577121e+01 4.827551e+04 2.197169e+02
##     coef.var
## 2.688482e-01
## ------------------------------------------------------------
## : BA44
## : sham
## : lex
##       median         mean      SE.mean CI.mean.0.95          var      std.dev
## 7.040000e+02 7.229303e+02 8.988271e+00 1.764482e+01 6.139965e+04 2.477895e+02
##     coef.var
## 3.427572e-01
## ------------------------------------------------------------
## : BA47
## : sham
## : lex
##       median         mean      SE.mean CI.mean.0.95          var      std.dev
## 7.000000e+02 7.336158e+02 9.116707e+00 1.789696e+01 6.316690e+04 2.513303e+02
##     coef.var
## 3.425911e-01
```

```
by(data$dur1, list(data$area, data$sham.real, data$gram.lex), stat.desc, basic=FALSE)
```

```
## : BA44
## : real
## : gram
##       median         mean      SE.mean CI.mean.0.95          var      std.dev
##  170.0000000  190.9607895    3.3412040    6.5590989 8484.3695277   92.1106374
##     coef.var
##    0.4823537
## ------------------------------------------------------------
## : BA47
## : real
## : gram
##       median         mean      SE.mean CI.mean.0.95          var      std.dev
##  185.0000000  201.8263158    3.4287079    6.7308774 8934.5890299   94.5229550
##     coef.var
##    0.4683381
## ------------------------------------------------------------
## : BA44
## : sham
## : gram
##       median         mean      SE.mean CI.mean.0.95          var      std.dev
##  197.0000000  208.6118421    3.2089060    6.2993855 7825.7793062   88.4634349
##     coef.var
##    0.4240576
## ------------------------------------------------------------
## : BA47
## : sham
## : gram
##       median         mean      SE.mean CI.mean.0.95          var      std.dev
## 1.875000e+02 2.112513e+02 3.868999e+00 7.595211e+00 1.137656e+04 1.066609e+02
##     coef.var
## 5.049007e-01
## ------------------------------------------------------------
```

```
## : BA44
## : real
## : lex
##        median          mean       SE.mean CI.mean.0.95          var      std.dev
## 2.230000e+02 2.466184e+02 5.412558e+00 1.062536e+01 2.226479e+04 1.492139e+02
##      coef.var
## 6.050396e-01
## ------------------------------------------------------------
## : BA47
## : real
## : lex
##        median          mean       SE.mean CI.mean.0.95          var      std.dev
##   233.0000000   244.3750000     3.2280141     6.3368965 7919.2570817   88.9902078
##      coef.var
##     0.3641543
## ------------------------------------------------------------
## : BA44
## : sham
## : lex
##        median          mean       SE.mean CI.mean.0.95          var      std.dev
##   237.0000000   241.8921053     2.8718042     5.6376228 6267.9171971   79.1701787
##      coef.var
##     0.3272954
## ------------------------------------------------------------
## : BA47
## : sham
## : lex
##        median          mean       SE.mean CI.mean.0.95          var      std.dev
##   238.0000000   246.4894737     2.6432510     5.1889514 5309.9498232   72.8694025
##      coef.var
##     0.2956289
```

```
by(data$dur2, list(data$area, data$sham.real, data$gram.lex), stat.desc, basic=FALSE)
```

```
## : BA44
## : real
## : gram
##        median          mean       SE.mean CI.mean.0.95          var      std.dev
## 7.350000e+02 7.390658e+02 4.826551e+00 9.474976e+00 1.770465e+04 1.330588e+02
##      coef.var
## 1.800365e-01
## ------------------------------------------------------------
## : BA47
## : real
## : gram
##        median          mean       SE.mean CI.mean.0.95          var      std.dev
## 7.340000e+02 7.382316e+02 5.566045e+00 1.092667e+01 2.354545e+04 1.534453e+02
##      coef.var
## 2.078552e-01
## ------------------------------------------------------------
## : BA44
## : sham
## : gram
##        median          mean       SE.mean CI.mean.0.95          var      std.dev
## 7.600000e+02 7.720829e+02 6.688247e+00 1.312966e+01 3.399681e+04 1.843822e+02
```

```
##     coef.var
## 2.388115e-01
## ------------------------------------------------------------
## : BA47
## : sham
## : gram
##       median         mean      SE.mean CI.mean.0.95          var      std.dev
## 7.425000e+02 7.637789e+02 6.733192e+00 1.321789e+01 3.445527e+04 1.856213e+02
##     coef.var
## 2.430301e-01
## ------------------------------------------------------------
## : BA44
## : real
## : lex
##       median         mean      SE.mean CI.mean.0.95          var      std.dev
## 6.955000e+02 7.115855e+02 5.656648e+00 1.110453e+01 2.431823e+04 1.559430e+02
##     coef.var
## 2.191487e-01
## ------------------------------------------------------------
## : BA47
## : real
## : lex
##       median         mean      SE.mean CI.mean.0.95          var      std.dev
## 7.015000e+02 7.122042e+02 6.366345e+00 1.249885e+01 2.918185e+04 1.708270e+02
##     coef.var
## 2.398567e-01
## ------------------------------------------------------------
## : BA44
## : sham
## : lex
##       median         mean      SE.mean CI.mean.0.95          var      std.dev
## 7.380000e+02 7.436368e+02 4.614881e+00 9.059448e+00 1.618582e+04 1.272235e+02
##     coef.var
## 1.710828e-01
## ------------------------------------------------------------
## : BA47
## : sham
## : lex
##       median         mean      SE.mean CI.mean.0.95          var      std.dev
## 7.260000e+02 7.318776e+02 5.138265e+00 1.008690e+01 2.006534e+04 1.416522e+02
##     coef.var
## 1.935463e-01
```

```
by(target$accuracy, list(target$target_filler, target$gram.lex), stat.desc, basic=FALSE)
```

```
## : filler
## : gram
## NULL
## ------------------------------------------------------------
## : target
## : gram
##       median         mean      SE.mean CI.mean.0.95          var      std.dev
##            1            1            0            0            0            0
##     coef.var
##            0
```

```
## ------------------------------------------------------------
## : filler
## : lex
## NULL
## ------------------------------------------------------------
## : target
## : lex
##       median         mean      SE.mean CI.mean.0.95          var      std.dev
##            1            1            0            0            0            0
##     coef.var
##            0
```

```r
# statistical models
library(lme4)
```

```
## Loading required package: Matrix

##
## Attaching package: 'Matrix'

## The following objects are masked from 'package:tidyr':
##
##     expand, pack, unpack
```

```r
library(lmerTest)
```

```
##
## Attaching package: 'lmerTest'

## The following object is masked from 'package:lme4':
##
##     lmer

## The following object is masked from 'package:stats':
##
##     step
```

```r
library(emmeans)
```

```
## Welcome to emmeans.
## NOTE -- Important change from versions <= 1.41:
##     Indicator predictors are now treated as 2-level factors by default.
##     To revert to old behavior, use emm_options(cov.keep = character(0))
```

```r
# RT as a dependent variable and 100K iterations
# full model with random slopes
modelrt=lmer(rt~area*gram.lex*sham.real+(1|participant)+(1|item) + (1 + area|participant)+(1 + gram.lex
```

```
## Warning in checkConv(attr(opt, "derivs"), opt$par, ctrl = control$checkConv, :
## unable to evaluate scaled gradient

## Warning in checkConv(attr(opt, "derivs"), opt$par, ctrl = control$checkConv, :
## Model failed to converge: degenerate Hessian with 1 negative eigenvalues
```

```r
# the model doesn't converge. Removing one random slope at a time.
modelrt=lmer(rt~area*gram.lex*sham.real+(1|participant)+(1|item) + (1 + area|participant)+(1 + sham.rea
```

```
## boundary (singular) fit: see ?isSingular

## Warning: Model failed to converge with 2 negative eigenvalues: -1.9e-05 -5.4e-01
```

```r
modelrt=lmer(rt~area*gram.lex*sham.real+(1|participant)+(1|item) + (1 + sham.real|participant), data=ta
```

```
## Warning in checkConv(attr(opt, "derivs"), opt$par, ctrl = control$checkConv, : Model is nearly unide
##  - Rescale variables?

## Warning: Model failed to converge with 1 negative eigenvalue: -1.1e-04
```

```r
modelrt=lmer(rt~session + area*gram.lex*sham.real+(1|participant)+(1|item), data=target, control=lmerCo

# model diagnostics
# residual plots for normality check. The right tail does not seem to be well fitted. log transformatio
plot(fitted(modelrt),residuals(modelrt))
```

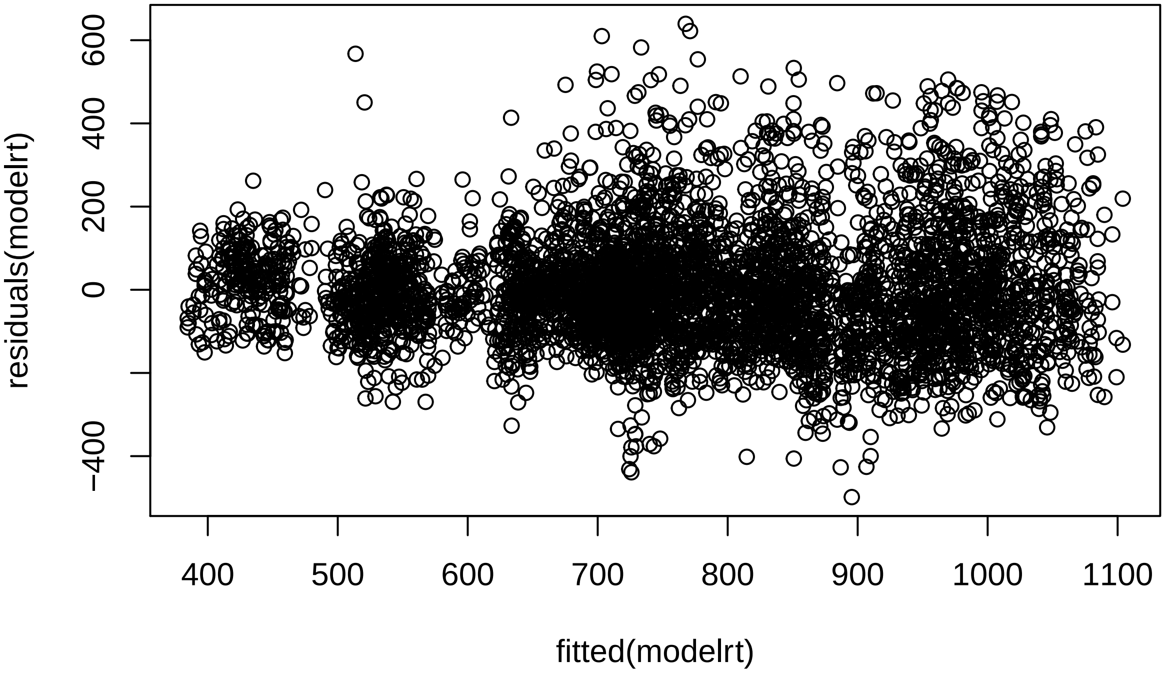


```r
qqnorm(residuals(modelrt)); qqline (residuals(modelrt))
```

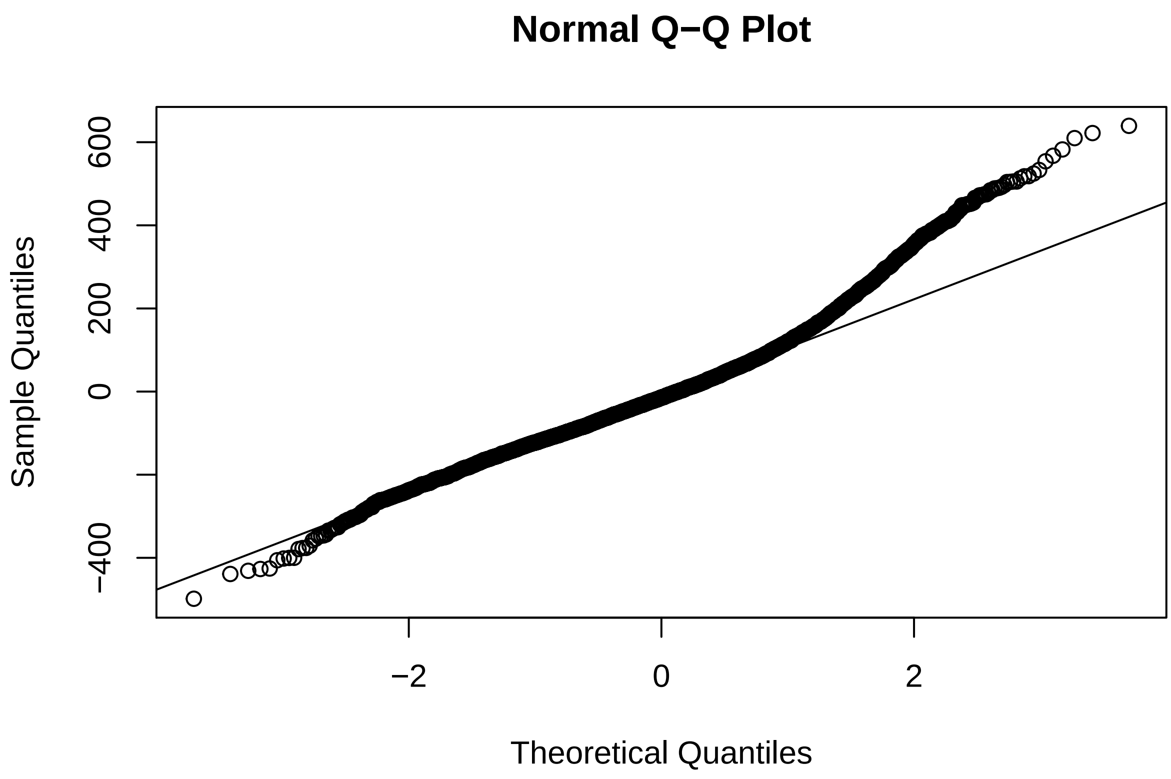


```
# Repeating the same procedure with log-transformation. full model with random slopes
modellogrt=lmer(log(rt)~area*gram.lex*sham.real+(1|participant)+(1|item) + (1 + area|participant)+(1 + g
```

```
## Warning in checkConv(attr(opt, "derivs"), opt$par, ctrl = control$checkConv, : Model is nearly uniden
##  - Rescale variables?
```

```
## Warning: Model failed to converge with 1 negative eigenvalue: -3.6e-05
```

```
# the model doesn't converge. Removing one random slope at a time.
modellogrt=lmer(log(rt)~area*gram.lex*sham.real+(1|participant)+(1|item) + (1 + area|participant)+(1 + s
```

```
## Warning in checkConv(attr(opt, "derivs"), opt$par, ctrl = control$checkConv, : Model is nearly uniden
##  - Rescale variables?
```

```
modellogrt=lmer(log(rt)~area*gram.lex*sham.real+(1|participant)+(1|item) + (1 + sham.real|participant),
```

```
## Warning in checkConv(attr(opt, "derivs"), opt$par, ctrl = control$checkConv, : Model is nearly uniden
##  - Rescale variables?
```

```
## Warning: Model failed to converge with 1 negative eigenvalue: -6.9e-05
```

```
# converged
modellogrt=lmer(log(rt)~sham.real*area*gram.lex+(1|participant)+(1|item), data=target, control=lmerCont

# additional model with session
modellogrt_session=lmer(log(rt)~session + sham.real*area*gram.lex+(1|participant)+(1|item), data=target
summary(modellogrt_session)
```

```
## Linear mixed model fit by REML. t-tests use Satterthwaite's method [
## lmerModLmerTest]
## Formula: log(rt) ~ session + sham.real * area * gram.lex + (1 | participant) +
##     (1 | item)
##    Data: target
```

```
## Control: lmerControl(optimizer = "bobyqa", optCtrl = list(maxfun = 1e+05))
##
## REML criterion at convergence: -2857.1
##
## Scaled residuals:
##     Min      1Q  Median      3Q     Max
## -5.0534 -0.6410 -0.0312  0.6094  4.2269
##
## Random effects:
##  Groups      Name        Variance  Std.Dev.
##  participant (Intercept) 0.0506516 0.22506
##  item        (Intercept) 0.0001975 0.01405
##  Residual                0.0305032 0.17465
## Number of obs: 4670, groups:  participant, 19; item, 16
##
## Fixed effects:
##                                     Estimate Std. Error         df t value
## (Intercept)                        6.694e+00  5.231e-02  1.888e+01 127.957
## session2                          -1.087e-01  5.291e-03  4.629e+03 -20.539
## sham.realsham                     -4.779e-02  1.019e-02  4.628e+03  -4.690
## areaBA47                           7.933e-02  1.022e-02  4.628e+03   7.760
## gram.lexlex                       -2.572e-02  1.026e-02  4.629e+03  -2.507
## sham.realsham:areaBA47            -8.417e-02  1.444e-02  4.628e+03  -5.829
## sham.realsham:gram.lexlex         -3.576e-02  1.444e-02  4.628e+03  -2.477
## areaBA47:gram.lexlex              -2.058e-02  1.451e-02  4.629e+03  -1.418
## sham.realsham:areaBA47:gram.lexlex  4.824e-02  2.045e-02  4.628e+03   2.359
##                                   Pr(>|t|)
## (Intercept)                        < 2e-16 ***
## session2                           < 2e-16 ***
## sham.realsham                     2.81e-06 ***
## areaBA47                          1.04e-14 ***
## gram.lexlex                         0.0122 *
## sham.realsham:areaBA47            5.94e-09 ***
## sham.realsham:gram.lexlex           0.0133 *
## areaBA47:gram.lexlex                0.1561
## sham.realsham:areaBA47:gram.lexlex   0.0184 *
## ---
## Signif. codes:  0 '***' 0.001 '**' 0.01 '*' 0.05 '.' 0.1 ' ' 1
##
## Correlation of Fixed Effects:
##             (Intr) sessn2 shm.rl arBA47 grm.lx sh.:BA47 shm.:. aBA47:
## session2    -0.049
## sham.relshm -0.095 -0.054
## areaBA47    -0.097 -0.001  0.500
## gram.lexlex -0.097  0.000  0.498  0.496
## shm.rl:BA47  0.069  0.001 -0.704 -0.708 -0.351
## shm.rlshm:.  0.069 -0.001 -0.703 -0.353 -0.710  0.496
## arBA47:grm.  0.068  0.006 -0.352 -0.704 -0.707  0.499    0.502
## shm.:BA47:. -0.048 -0.004  0.497  0.500  0.502 -0.706   -0.706 -0.710
```

```
# plotting the residuals. Looks better
plot(fitted(modellogrt),residuals(modellogrt))
```

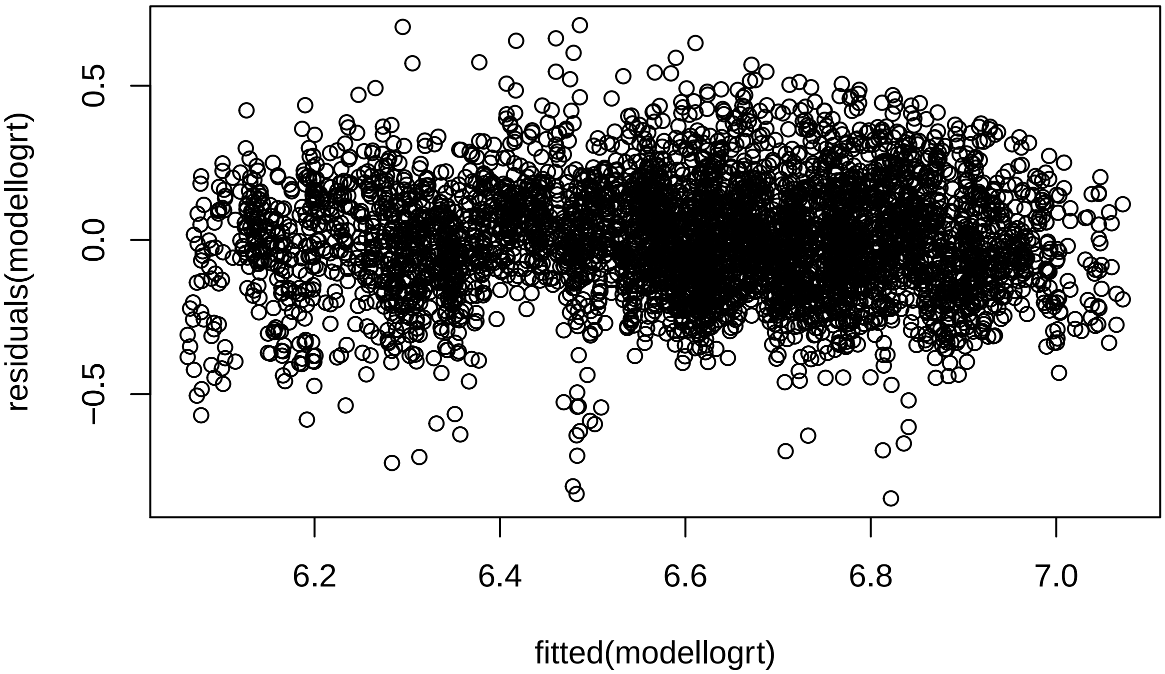


```r
qqnorm(residuals(modellogrt)); qqline (residuals(modellogrt))
```

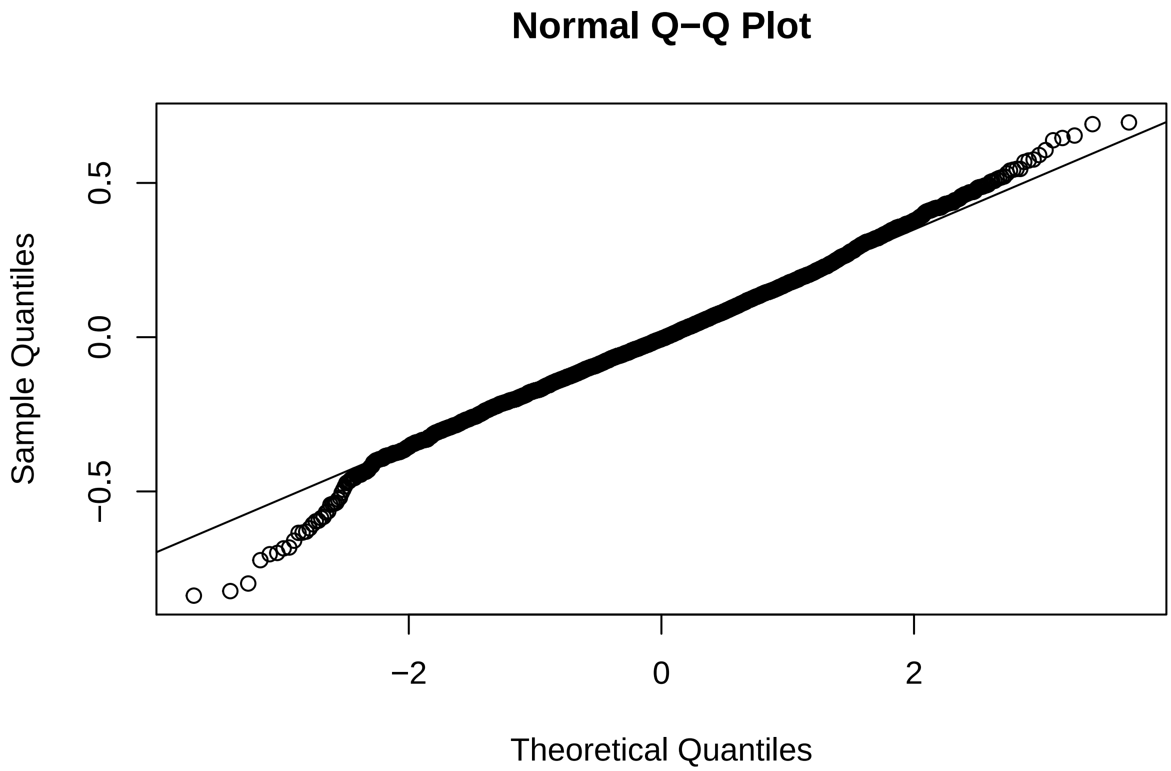


```r
qqnorm(residuals(modellogrt)); qqline (residuals(modellogrt))
```

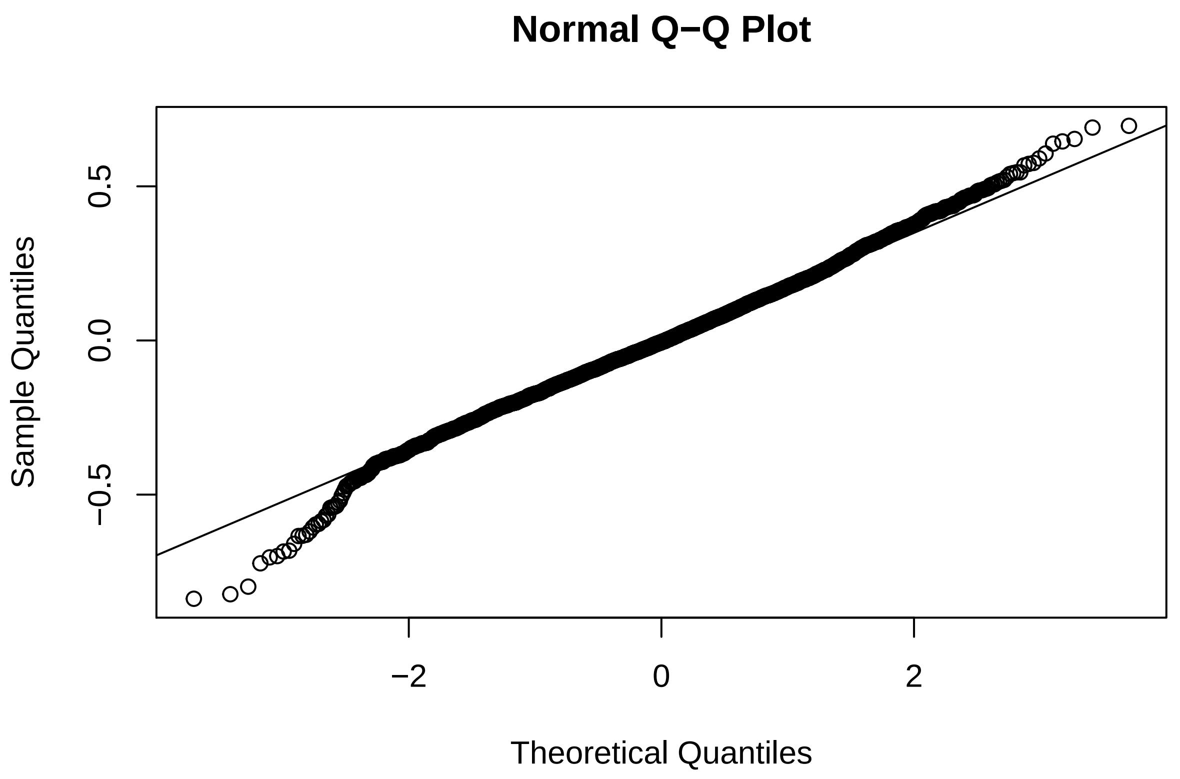


```r
# the best fitting model
step(modellogrt)
```

```
## Backward reduced random-effect table:
##
##                      Eliminated npar  logLik     AIC    LRT Df Pr(>Chisq)
## <none>                            11 1230.96 -2439.9
## (1 | participant)           0     10 -765.87  1551.8 3993.7  1  < 2.2e-16 ***
## (1 | item)                  0     10 1225.67 -2431.3   10.6  1   0.001146 **
## ---
## Signif. codes:  0 '***' 0.001 '**' 0.01 '*' 0.05 '.' 0.1 ' ' 1
##
## Backward reduced fixed-effect table:
## Degrees of freedom method: Satterthwaite
##
##                            Eliminated  Sum Sq Mean Sq NumDF  DenDF F value  Pr(>F)
## sham.real:area:gram.lex             0 0.15948 0.15948     1 4629.4  4.7925 0.02863
##
## sham.real:area:gram.lex *
## ---
## Signif. codes:  0 '***' 0.001 '**' 0.01 '*' 0.05 '.' 0.1 ' ' 1
##
## Model found:
## log(rt) ~ sham.real * area * gram.lex + (1 | participant) + (1 |
##     item)
```

```r
# obtaining model parameters of the best fitting model
summary(modellogrt)
```

```
## Linear mixed model fit by REML. t-tests use Satterthwaite's method [
```

```
## lmerModLmerTest]
## Formula: log(rt) ~ sham.real * area * gram.lex + (1 | participant) + (1 |
##     item)
##    Data: target
## Control: lmerControl(optimizer = "bobyqa", optCtrl = list(maxfun = 1e+05))
##
## REML criterion at convergence: -2461.9
##
## Scaled residuals:
##     Min      1Q  Median      3Q     Max
## -4.5928 -0.6441 -0.0308  0.6457  3.8163
##
## Random effects:
##  Groups      Name        Variance Std.Dev.
##  participant (Intercept) 0.049738 0.22302
##  item        (Intercept) 0.000195 0.01396
##  Residual                0.033276 0.18242
## Number of obs: 4670, groups:  participant, 19; item, 16
##
## Fixed effects:
##                                     Estimate Std. Error         df t value
## (Intercept)                          6.64174    0.05183   18.85671 128.133
## sham.realsham                       -0.05918    0.01063 4629.20204  -5.569
## areaBA47                             0.07902    0.01068 4629.52990   7.401
## gram.lexlex                         -0.02580    0.01071 4629.58265  -2.408
## sham.realsham:areaBA47              -0.08389    0.01508 4629.35796  -5.563
## sham.realsham:gram.lexlex           -0.03592    0.01508 4629.26371  -2.382
## areaBA47:gram.lexlex                -0.01880    0.01516 4629.88896  -1.240
## sham.realsham:areaBA47:gram.lexlex   0.04676    0.02136 4629.43655   2.189
##                                   Pr(>|t|)
## (Intercept)                        < 2e-16 ***
## sham.realsham                     2.71e-08 ***
## areaBA47                          1.60e-13 ***
## gram.lexlex                         0.0161 *
## sham.realsham:areaBA47            2.80e-08 ***
## sham.realsham:gram.lexlex           0.0173 *
## areaBA47:gram.lexlex                0.2149
## sham.realsham:areaBA47:gram.lexlex  0.0286 *
## ---
## Signif. codes:  0 '***' 0.001 '**' 0.01 '*' 0.05 '.' 0.1 ' ' 1
##
## Correlation of Fixed Effects:
##             (Intr) shm.rl arBA47 grm.lx sh.:BA47 shm.:. aBA47:
## sham.relshm -0.103
## areaBA47    -0.103  0.501
## gram.lexlex -0.102  0.499  0.496
## shm.rl:BA47  0.073 -0.705 -0.708 -0.351
## shm.rlshm:.  0.073 -0.704 -0.353 -0.710  0.496
## arBA47:grm.  0.072 -0.352 -0.704 -0.707  0.499    0.502
## shm.:BA47:. -0.051  0.497  0.500  0.502 -0.706   -0.706 -0.710
```

```
# estimated marginal means
model.emm <- emmeans(modellogrt, ~ gram.lex * area * sham.real, pbkrtest.limit = 4670)
contrast(model.emm, "consec", simple = "each", combine = TRUE, adjust = "mvt")
```

```
##  area sham.real gram.lex contrast    estimate     SE   df t.ratio p.value
##  BA44 real      .        lex - gram  -0.02580 0.0107 4630  -2.408 0.1469
##  BA47 real      .        lex - gram  -0.04460 0.0107 4630  -4.160 0.0004
##  BA44 sham      .        lex - gram  -0.06171 0.0106 4629  -5.815 <.0001
##  BA47 sham      .        lex - gram  -0.03376 0.0107 4629  -3.163 0.0169
##  .    real      gram     BA47 - BA44  0.07902 0.0107 4630   7.401 <.0001
##  .    real      lex      BA47 - BA44  0.06022 0.0108 4630   5.597 <.0001
##  .    sham      gram     BA47 - BA44 -0.00487 0.0106 4629  -0.457 0.9995
##  .    sham      lex      BA47 - BA44  0.02309 0.0106 4629   2.171 0.2475
##  BA44 .         gram     sham - real -0.05918 0.0106 4629  -5.569 <.0001
##  BA44 .         lex      sham - real -0.09509 0.0107 4630  -8.885 <.0001
##  BA47 .         gram     sham - real -0.14307 0.0107 4630 -13.369 <.0001
##  BA47 .         lex      sham - real -0.13223 0.0107 4630 -12.366 <.0001
##
## Degrees-of-freedom method: kenward-roger
## Results are given on the log (not the response) scale.
## P value adjustment: mvt method for 12 tests
```

```
# best fitting model summary
summary(modellogrt)
```

```
## Linear mixed model fit by REML. t-tests use Satterthwaite's method [
## lmerModLmerTest]
## Formula: log(rt) ~ sham.real * area * gram.lex + (1 | participant) + (1 |
##     item)
##    Data: target
## Control: lmerControl(optimizer = "bobyqa", optCtrl = list(maxfun = 1e+05))
##
## REML criterion at convergence: -2461.9
##
## Scaled residuals:
##     Min      1Q  Median      3Q     Max
## -4.5928 -0.6441 -0.0308  0.6457  3.8163
##
## Random effects:
##  Groups      Name        Variance Std.Dev.
##  participant (Intercept) 0.049738 0.22302
##  item        (Intercept) 0.000195 0.01396
##  Residual                0.033276 0.18242
## Number of obs: 4670, groups:  participant, 19; item, 16
##
## Fixed effects:
##                                    Estimate Std. Error         df t value
## (Intercept)                         6.64174    0.05183   18.85671 128.133
## sham.realsham                      -0.05918    0.01063 4629.20204  -5.569
## areaBA47                            0.07902    0.01068 4629.52990   7.401
## gram.lexlex                        -0.02580    0.01071 4629.58265  -2.408
## sham.realsham:areaBA47             -0.08389    0.01508 4629.35796  -5.563
## sham.realsham:gram.lexlex          -0.03592    0.01508 4629.26371  -2.382
## areaBA47:gram.lexlex               -0.01880    0.01516 4629.88896  -1.240
## sham.realsham:areaBA47:gram.lexlex  0.04676    0.02136 4629.43655   2.189
##                                    Pr(>|t|)
## (Intercept)                         < 2e-16 ***
## sham.realsham                      2.71e-08 ***
## areaBA47                           1.60e-13 ***
```

```
## gram.lexlex                          0.0161 *
## sham.realsham:areaBA47             2.80e-08 ***
## sham.realsham:gram.lexlex            0.0173 *
## areaBA47:gram.lexlex                 0.2149
## sham.realsham:areaBA47:gram.lexlex   0.0286 *
## ---
## Signif. codes:  0 '***' 0.001 '**' 0.01 '*' 0.05 '.' 0.1 ' ' 1
##
## Correlation of Fixed Effects:
##             (Intr) shm.rl arBA47 grm.lx sh.:BA47 shm.:. aBA47:
## sham.relshm -0.103
## areaBA47    -0.103  0.501
## gram.lexlex -0.102  0.499  0.496
## shm.rl:BA47  0.073 -0.705 -0.708 -0.351
## shm.rlshm:.  0.073 -0.704 -0.353 -0.710  0.496
## arBA47:grm.  0.072 -0.352 -0.704 -0.707  0.499    0.502
## shm.:BA47:. -0.051  0.497  0.500  0.502 -0.706   -0.706 -0.710
```

```r
# filler analysis, only RTs
filler = subset(data, target_filler == "filler" & accuracy > 0)
filler_up = mean(filler$rt) + 3*sd(filler$rt)
filler = filler %>% drop_na(rt)
sum(filler$rt > filler_up)
```

```
## [1] 12
```

```r
filler = subset(filler, rt < filler_up)

# filler model
# RT as a dependent variable and 100K iterations
# full model with random slopes
modelf=lmer(log(rt)~area*gram.lex*sham.real+(1|participant)+(1|item) + (1 + area|participant)+(1 + gram
```

```
## Warning in checkConv(attr(opt, "derivs"), opt$par, ctrl = control$checkConv, : Model is nearly unide
##  - Rescale variables?

## Warning: Model failed to converge with 2 negative eigenvalues: -1.5e-05 -2.6e-05
```

```r
# the model doesn't converge. Removing one random slope at a time.
modelf=lmer(log(rt)~area*gram.lex*sham.real+(1|participant)+(1|item) + (1 + area|participant)+(1 + sham
```

```
## Warning in checkConv(attr(opt, "derivs"), opt$par, ctrl = control$checkConv, : Model is nearly unide
##  - Rescale variables?

## Warning: Model failed to converge with 2 negative eigenvalues: -8.3e-06 -1.4e-05
```

```r
modelf=lmer(log(rt)~area*gram.lex*sham.real+(1|participant)+(1|item) + (1 + sham.real|participant), data


# singular. issue with item random effect
modelf=lmer(log(rt)~area*gram.lex*sham.real+(1|participant)+(1|item), data=filler, control=lmerControl(o
```

```
## boundary (singular) fit: see ?isSingular
```

```r
modelf=lmer(log(rt)~area*gram.lex*sham.real+(1|participant), data=filler, control=lmerControl(optimizer

# plotting the residuals. Looks better
plot(fitted(modelf),residuals(modelf))
```

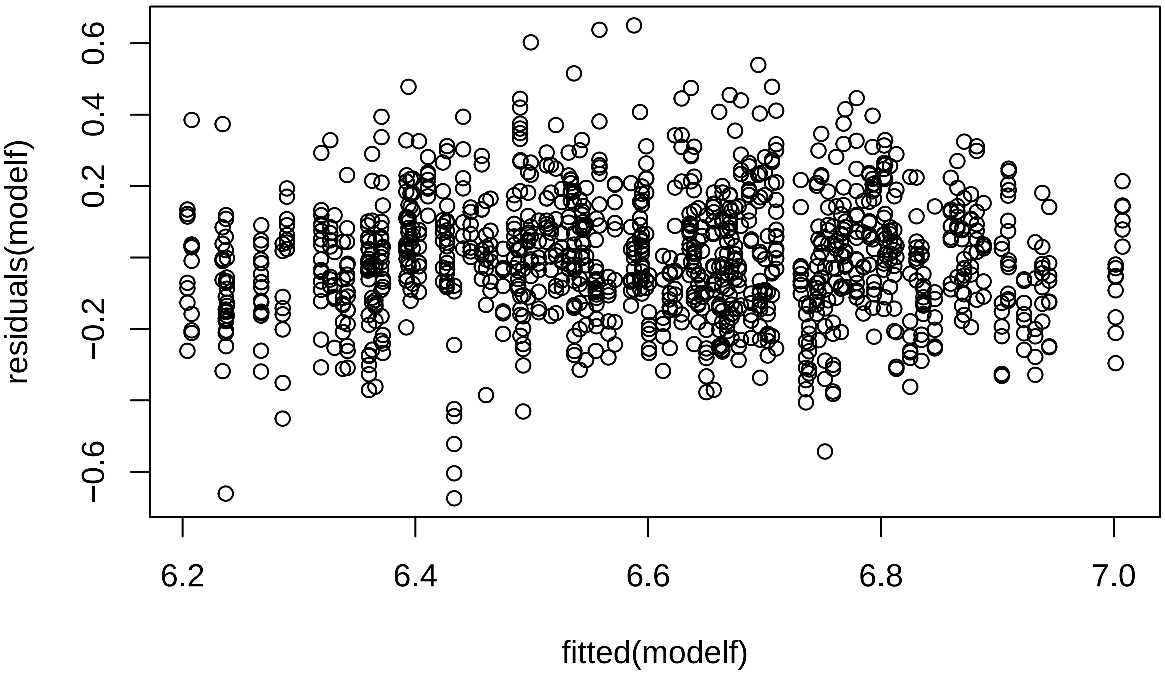


```
qqnorm(residuals(modelf)); qqline (residuals(modelf))
```

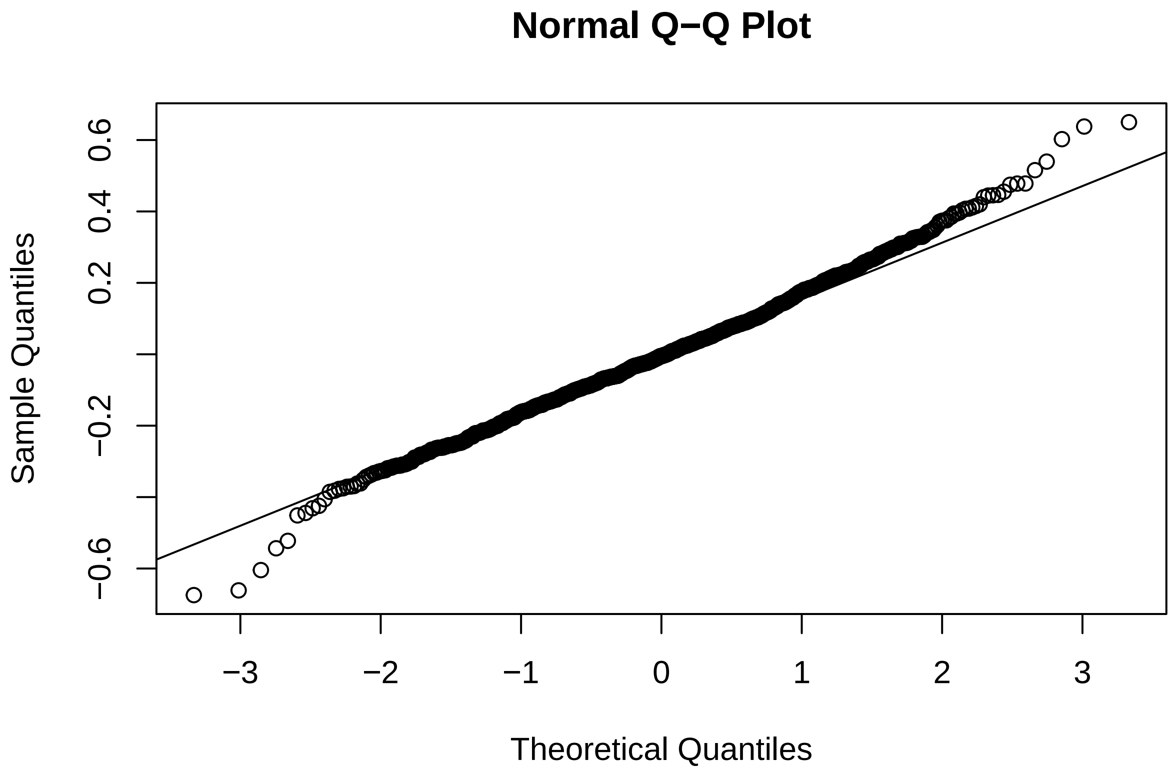


```
# the step function suggest removing task as a fixed effect variable. However, we keep it with three fi
step(modelf)
```

```
## Backward reduced random-effect table:
##
##                       Eliminated npar  logLik     AIC     LRT Df Pr(>Chisq)
```

```
## <none>                          10 306.364 -592.73
## (1 | participant)          0    9 -57.898  133.80 728.52  1  < 2.2e-16 ***
## ---
## Signif. codes:  0 '***' 0.001 '**' 0.01 '*' 0.05 '.' 0.1 ' ' 1
##
## Backward reduced fixed-effect table:
## Degrees of freedom method: Satterthwaite
##
##                         Eliminated Sum Sq Mean Sq NumDF DenDF  F value
## area:gram.lex:sham.real          1 0.0160  0.0160     1  1131   0.5147
## area:gram.lex                    2 0.0016  0.0016     1  1132   0.0506
## gram.lex:sham.real               3 0.0073  0.0073     1  1133   0.2343
## gram.lex                         0 3.3738  3.3738     1  1134 108.8040
## area:sham.real                   0 0.5666  0.5666     1  1134  18.2733
##                            Pr(>F)
## area:gram.lex:sham.real    0.4733
## area:gram.lex              0.8221
## gram.lex:sham.real         0.6284
## gram.lex                < 2.2e-16 ***
## area:sham.real          2.075e-05 ***
## ---
## Signif. codes:  0 '***' 0.001 '**' 0.01 '*' 0.05 '.' 0.1 ' ' 1
##
## Model found:
## log(rt) ~ area + gram.lex + sham.real + (1 | participant) + area:sham.real
```

```r
summary(modelf)
```

```
## Linear mixed model fit by REML. t-tests use Satterthwaite's method [
## lmerModLmerTest]
## Formula: log(rt) ~ area * gram.lex * sham.real + (1 | participant)
##    Data: filler
## Control: lmerControl(optimizer = "bobyqa", optCtrl = list(maxfun = 1e+05))
##
## REML criterion at convergence: -612.7
##
## Scaled residuals:
##     Min      1Q  Median      3Q     Max
## -3.8262 -0.6324 -0.0268  0.5810  3.6875
##
## Random effects:
##  Groups      Name        Variance Std.Dev.
##  participant (Intercept) 0.03359  0.1833
##  Residual                0.03107  0.1763
## Number of obs: 1157, groups:  participant, 19
##
## Fixed effects:
##                                    Estimate Std. Error         df t value
## (Intercept)                       6.672e+00  4.448e-02  2.185e+01 150.011
## areaBA47                          6.296e-02  2.064e-02  1.131e+03   3.050
## gram.lexlex                      -1.081e-01  2.064e-02  1.131e+03  -5.237
## sham.realsham                    -5.638e-02  2.053e-02  1.131e+03  -2.746
## areaBA47:gram.lexlex              1.027e-02  2.939e-02  1.131e+03   0.350
## areaBA47:sham.realsham           -7.374e-02  2.921e-02  1.131e+03  -2.524
## gram.lexlex:sham.realsham         4.794e-03  2.924e-02  1.131e+03   0.164
```

```
## areaBA47:gram.lexlex:sham.realsham -2.975e-02  4.147e-02  1.131e+03  -0.717
##                                     Pr(>|t|)
## (Intercept)                          < 2e-16 ***
## areaBA47                             0.00234 **
## gram.lexlex                         1.95e-07 ***
## sham.realsham                        0.00613 **
## areaBA47:gram.lexlex                 0.72672
## areaBA47:sham.realsham               0.01173 *
## gram.lexlex:sham.realsham            0.86980
## areaBA47:gram.lexlex:sham.realsham   0.47327
## ---
## Signif. codes:  0 '***' 0.001 '**' 0.01 '*' 0.05 '.' 0.1 ' ' 1
##
## Correlation of Fixed Effects:
##             (Intr) arBA47 grm.lx shm.rl arBA47:g. arBA47:s. grm.:.
## areaBA47    -0.229
## gram.lexlex -0.229  0.492
## sham.relshm -0.230  0.496  0.495
## arBA47:grm.  0.161 -0.702 -0.702 -0.348
## arBA47:shm.  0.162 -0.707 -0.348 -0.703  0.496
## grm.lxlx:s.  0.161 -0.347 -0.706 -0.702  0.495     0.493
## arBA47:g.:. -0.114  0.497  0.498  0.495 -0.709    -0.704    -0.705
```

```
# estimated marginal means
model.emmf <- emmeans(modelf, ~ gram.lex * area * sham.real)
contrast(model.emmf, "consec", simple = "each", combine = TRUE, adjust = "mvt")
```

```
##  area sham.real gram.lex contrast    estimate     SE   df t.ratio p.value
##  BA44 real      .        lex - gram   -0.1081 0.0206 1131 -5.237  <.0001
##  BA47 real      .        lex - gram   -0.0978 0.0209 1131 -4.672  <.0001
##  BA44 sham      .        lex - gram   -0.1033 0.0207 1131 -4.987  <.0001
##  BA47 sham      .        lex - gram   -0.1228 0.0207 1131 -5.938  <.0001
##  .    real      gram     BA47 - BA44   0.0630 0.0206 1131  3.050  0.0252
##  .    real      lex      BA47 - BA44   0.0732 0.0209 1131  3.497  0.0055
##  .    sham      gram     BA47 - BA44  -0.0108 0.0207 1131 -0.521  0.9988
##  .    sham      lex      BA47 - BA44  -0.0303 0.0207 1131 -1.461  0.7220
##  BA44 .         gram     sham - real  -0.0564 0.0205 1131 -2.746  0.0612
##  BA44 .         lex      sham - real  -0.0516 0.0208 1131 -2.476  0.1247
##  BA47 .         gram     sham - real  -0.1301 0.0208 1131 -6.263  <.0001
##  BA47 .         lex      sham - real  -0.1551 0.0208 1131 -7.448  <.0001
##
## Degrees-of-freedom method: kenward-roger
## Results are given on the log (not the response) scale.
## P value adjustment: mvt method for 12 tests
```

```
# Dur1 as a dependent variable and 100K iterations
# full model with random slopes
modeldur1=lmer(dur1~area*gram.lex*sham.real+(1|participant)+(1|item) + (1 + area|participant)+(1 + gram
```

```
## Warning in checkConv(attr(opt, "derivs"), opt$par, ctrl = control$checkConv, :
## unable to evaluate scaled gradient

## Warning in checkConv(attr(opt, "derivs"), opt$par, ctrl = control$checkConv, :
## Model failed to converge: degenerate Hessian with 1 negative eigenvalues

## Warning: Model failed to converge with 3 negative eigenvalues: -1.5e-05 -4.4e-05
## -1.5e-04
```

```r
# no warning message
modeldur1=lmer(dur1~area*gram.lex*sham.real+(1|participant)+(1|item) + (1 + area|participant)+(1 + sham

# warning message about convergence. further reducing
step(modeldur1)
```

```
## Warning: Model failed to converge with 2 negative eigenvalues: -3.3e-05 -1.3e-04

## Warning in checkConv(attr(opt, "derivs"), opt$par, ctrl = control$checkConv, :
## unable to evaluate scaled gradient

## Warning in checkConv(attr(opt, "derivs"), opt$par, ctrl = control$checkConv, :
## Model failed to converge: degenerate Hessian with 1 negative eigenvalues

## Backward reduced random-effect table:
##
##                                              Eliminated npar logLik   AIC    LRT
## <none>                                                    17 -23924 47881
## (1 | participant)                                     1   16 -23924 47879   0.00
## (1 | item)                                            0   15 -24054 48139 261.92
## area in (1 + area | participant)                      0   14 -23978 47983 108.11
## sham.real in (1 + sham.real | participant)            0   14 -24287 48602 727.10
##                                              Df Pr(>Chisq)
## <none>
## (1 | participant)                             1          1
## (1 | item)                                    1     <2e-16 ***
## area in (1 + area | participant)              2     <2e-16 ***
## sham.real in (1 + sham.real | participant)    2     <2e-16 ***
## ---
## Signif. codes:  0 '***' 0.001 '**' 0.01 '*' 0.05 '.' 0.1 ' ' 1
##
## Backward reduced fixed-effect table:
## Degrees of freedom method: Satterthwaite
##
##                         Eliminated Sum Sq Mean Sq NumDF  DenDF F value
## area:gram.lex:sham.real          0  26286   26286     1 4593.3  16.738
##                            Pr(>F)
## area:gram.lex:sham.real 4.366e-05 ***
## ---
## Signif. codes:  0 '***' 0.001 '**' 0.01 '*' 0.05 '.' 0.1 ' ' 1
##
## Model found:
## dur1 ~ area + gram.lex + sham.real + (1 | item) + (1 + area |
##     participant) + (1 + sham.real | participant) + area:gram.lex +
##     area:sham.real + gram.lex:sham.real + area:gram.lex:sham.real
```

```r
modeldur1=lmer(dur1~area*gram.lex*sham.real+(1|participant)+(1|item) + (1 + area|participant), data=tar

# warning message about convergence again. further reducing
step(modeldur1)
```

```
## Warning in checkConv(attr(opt, "derivs"), opt$par, ctrl = control$checkConv, :
## unable to evaluate scaled gradient

## Warning in checkConv(attr(opt, "derivs"), opt$par, ctrl = control$checkConv, :
## Model failed to converge: degenerate Hessian with 1 negative eigenvalues
```

```
## Warning: Model failed to converge with 1 negative eigenvalue: -1.7e-05

## Backward reduced random-effect table:
##
##                                    Eliminated npar logLik   AIC     LRT Df
## <none>                                          14 -24287 48602
## (1 | participant)                           1   13 -24287 48600   0.000  1
## (1 | item)                                  0   12 -24392 48809 210.444  1
## area in (1 + area | participant)            0   11 -24324 48669  72.907  2
##                                    Pr(>Chisq)
## <none>
## (1 | participant)                           1
## (1 | item)                            <2e-16 ***
## area in (1 + area | participant)      <2e-16 ***
## ---
## Signif. codes:  0 '***' 0.001 '**' 0.01 '*' 0.05 '.' 0.1 ' ' 1
##
## Backward reduced fixed-effect table:
## Degrees of freedom method: Satterthwaite
##
##                           Eliminated Sum Sq Mean Sq NumDF  DenDF F value
## area:gram.lex:sham.real            0  24836   24836     1 4611.5  13.298
##                              Pr(>F)
## area:gram.lex:sham.real 0.0002686 ***
## ---
## Signif. codes:  0 '***' 0.001 '**' 0.01 '*' 0.05 '.' 0.1 ' ' 1
##
## Model found:
## dur1 ~ area + gram.lex + sham.real + (1 | item) + (1 + area |
##     participant) + area:gram.lex + area:sham.real + gram.lex:sham.real +
##     area:gram.lex:sham.real
```

```
modeldur1=lmer(dur1~area*gram.lex*sham.real+(1|participant)+(1|item), data=target, control=lmerControl(
step(modeldur1)
```

```
## Backward reduced random-effect table:
##
##                   Eliminated npar logLik   AIC     LRT Df Pr(>Chisq)
## <none>                          11 -24324 48669
## (1 | participant)          0   10 -25736 51493 2825.67  1  < 2.2e-16 ***
## (1 | item)                 0   10 -24425 48870  203.08  1  < 2.2e-16 ***
## ---
## Signif. codes:  0 '***' 0.001 '**' 0.01 '*' 0.05 '.' 0.1 ' ' 1
##
## Backward reduced fixed-effect table:
## Degrees of freedom method: Satterthwaite
##
##                           Eliminated Sum Sq Mean Sq NumDF  DenDF F value   Pr(>F)
## area:gram.lex:sham.real            0  24977   24977     1 4629.1  13.065 0.000304
##
## area:gram.lex:sham.real ***
## ---
## Signif. codes:  0 '***' 0.001 '**' 0.01 '*' 0.05 '.' 0.1 ' ' 1
##
## Model found:
```

```
## dur1 ~ area * gram.lex * sham.real + (1 | participant) + (1 |
##     item)
```

```r
# converged

summary(modeldur1)
```

```
## Linear mixed model fit by REML. t-tests use Satterthwaite's method [
## lmerModLmerTest]
## Formula: dur1 ~ area * gram.lex * sham.real + (1 | participant) + (1 |
##     item)
##    Data: target
## Control: lmerControl(optimizer = "bobyqa", optCtrl = list(maxfun = 1e+05))
##
## REML criterion at convergence: 48647.1
##
## Scaled residuals:
##     Min      1Q  Median      3Q     Max
## -3.2180 -0.6207 -0.0657  0.5319  9.7302
##
## Random effects:
##  Groups      Name        Variance Std.Dev.
##  participant (Intercept) 1774.1   42.12
##  item        (Intercept)  110.5   10.51
##  Residual                1911.7   43.72
## Number of obs: 4670, groups:  participant, 19; item, 16
##
## Fixed effects:
##                                     Estimate Std. Error       df t value
## (Intercept)                          164.720     10.176   21.780  16.188
## areaBA47                              14.158      2.559 4629.099   5.532
## gram.lexlex                           55.855      2.568 4629.102  21.749
## sham.realsham                         21.840      2.547 4629.045   8.574
## areaBA47:gram.lexlex                  -8.338      3.633 4629.155  -2.295
## areaBA47:sham.realsham               -16.752      3.615 4629.069  -4.634
## gram.lexlex:sham.realsham            -15.689      3.615 4629.049  -4.340
## areaBA47:gram.lexlex:sham.realsham    18.506      5.120 4629.082   3.615
##                                     Pr(>|t|)
## (Intercept)                         1.26e-13 ***
## areaBA47                            3.35e-08 ***
## gram.lexlex                          < 2e-16 ***
## sham.realsham                        < 2e-16 ***
## areaBA47:gram.lexlex                0.021769 *
## areaBA47:sham.realsham              3.68e-06 ***
## gram.lexlex:sham.realsham           1.45e-05 ***
## areaBA47:gram.lexlex:sham.realsham 0.000304 ***
## ---
## Signif. codes:  0 '***' 0.001 '**' 0.01 '*' 0.05 '.' 0.1 ' ' 1
##
## Correlation of Fixed Effects:
##             (Intr) arBA47 grm.lx shm.rl arBA47:g. arBA47:s. grm.:.
## areaBA47    -0.125
## gram.lexlex -0.125  0.496
## sham.relshm -0.126  0.501  0.499
## arBA47:grm.  0.088 -0.704 -0.707 -0.352
```

```
## arBA47:shm.  0.089 -0.708 -0.351 -0.705  0.499
## grm.lxlx:s.  0.089 -0.353 -0.710 -0.704  0.502     0.496
## arBA47:g.:. -0.063  0.500  0.502  0.497 -0.710    -0.706    -0.706
```

```
# residual plots for normality check. right skew. the same procedure again with logdur1
plot(fitted(modeldur1),residuals(modeldur1))
```

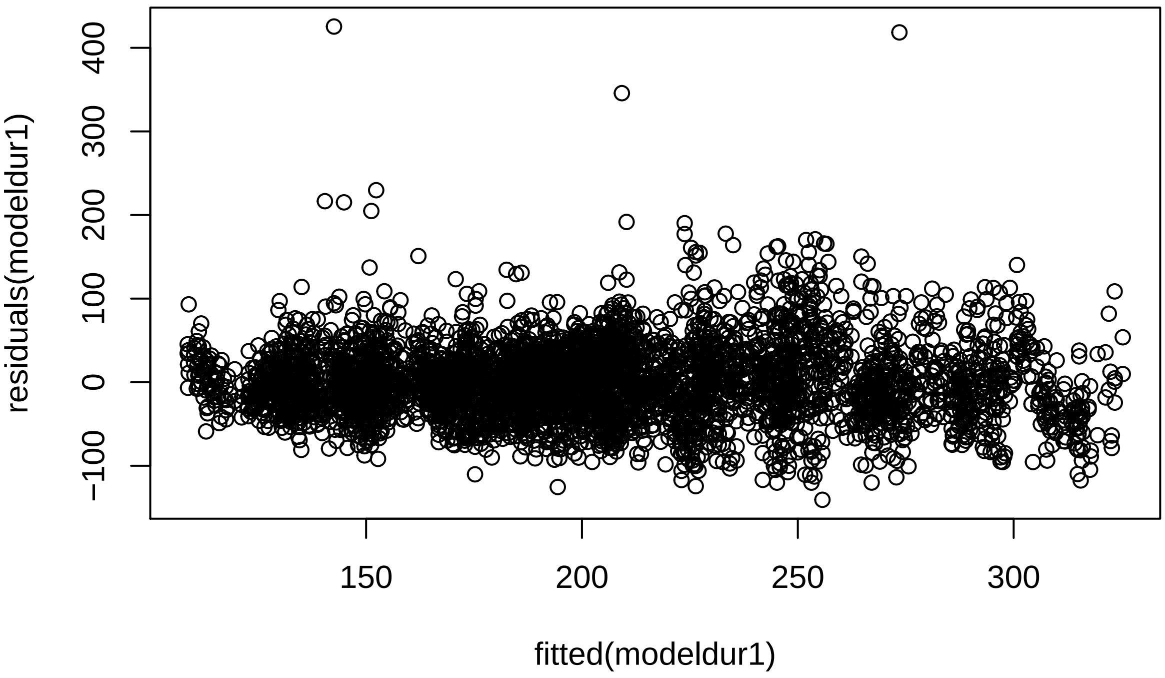


```
qqnorm(residuals(modeldur1)); qqline (residuals(modeldur1))
```

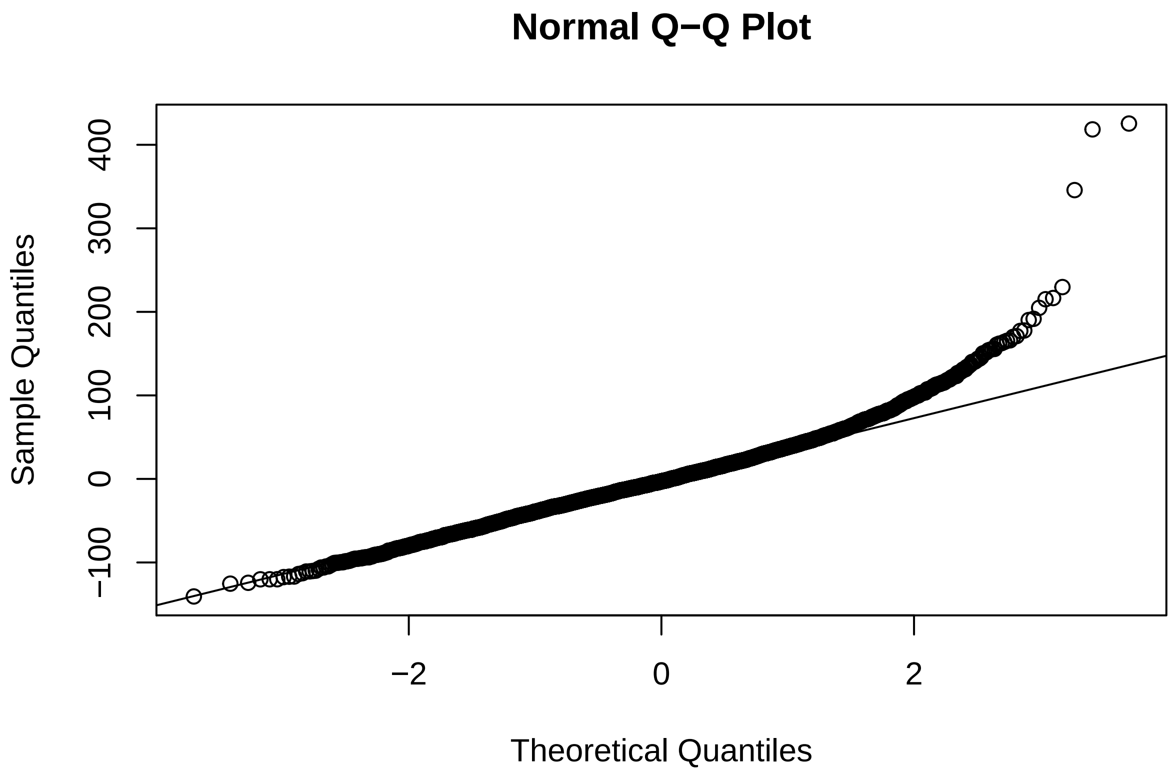

```r
# full model with random slopes
modeldur1=lmer(log(dur1)~area*gram.lex*sham.real+(1|participant)+(1|item) + (1 + area|participant)+(1 +
```

```
## Warning: Model failed to converge with 1 negative eigenvalue: -7.9e-05
```

```r
modeldur1=lmer(log(dur1)~area*gram.lex*sham.real+(1|participant)+(1|item) + (1 + area|participant)+(1 +
```

```
## Warning: Model failed to converge with 1 negative eigenvalue: -1.0e-05
```

```r
modeldur1=lmer(log(dur1)~area*gram.lex*sham.real+(1|participant)+(1|item) + (1 + area|participant), data
```

```
## Warning: Model failed to converge with 1 negative eigenvalue: -8.3e-06
```

```r
modeldur1=lmer(log(dur1)~area*gram.lex*sham.real+(1|participant)+(1|item), data=target, control=lmerCon
step(modeldur1)
```

```
## Backward reduced random-effect table:
##
##                     Eliminated npar  logLik     AIC     LRT Df Pr(>Chisq)
## <none>                           11  392.89 -763.78
## (1 | participant)           0    10 -980.23 1980.46 2746.25  1  < 2.2e-16 ***
## (1 | item)                  0    10  251.82 -483.64  282.15  1  < 2.2e-16 ***
## ---
## Signif. codes:  0 '***' 0.001 '**' 0.01 '*' 0.05 '.' 0.1 ' ' 1
##
## Backward reduced fixed-effect table:
## Degrees of freedom method: Satterthwaite
##
##                          Eliminated Sum Sq Mean Sq NumDF  DenDF F value
## area:gram.lex:sham.real           0 0.7291  0.7291     1 4629.1   15.37
##                             Pr(>F)
## area:gram.lex:sham.real 8.964e-05 ***
## ---
## Signif. codes:  0 '***' 0.001 '**' 0.01 '*' 0.05 '.' 0.1 ' ' 1
##
## Model found:
## log(dur1) ~ area * gram.lex * sham.real + (1 | participant) +
##     (1 | item)
```

```r
summary(modeldur1)
```

```
## Linear mixed model fit by REML. t-tests use Satterthwaite's method [
## lmerModLmerTest]
## Formula: log(dur1) ~ area * gram.lex * sham.real + (1 | participant) +
##     (1 | item)
##    Data: target
## Control: lmerControl(optimizer = "bobyqa", optCtrl = list(maxfun = 1e+05))
##
## REML criterion at convergence: -785.8
##
## Scaled residuals:
##     Min      1Q  Median      3Q     Max
## -4.5103 -0.6141  0.0328  0.6415  6.5176
##
## Random effects:
##  Groups      Name        Variance Std.Dev.
##  participant (Intercept) 0.042409 0.20593
```

```
##  item        (Intercept) 0.003704 0.06086
##  Residual                0.047435 0.21780
## Number of obs: 4670, groups:  participant, 19; item, 16
##
## Fixed effects:
##                                      Estimate Std. Error         df t value
## (Intercept)                           5.04658    0.05044   22.86029 100.045
## areaBA47                              0.08703    0.01275 4629.07748   6.826
## gram.lexlex                           0.31106    0.01279 4629.07755  24.315
## sham.realsham                         0.12052    0.01269 4629.03545   9.498
## areaBA47:gram.lexlex                 -0.05081    0.01810 4629.11959  -2.807
## areaBA47:sham.realsham               -0.09836    0.01801 4629.05351  -5.463
## gram.lexlex:sham.realsham            -0.08946    0.01801 4629.03669  -4.969
## areaBA47:gram.lexlex:sham.realsham    0.09999    0.02550 4629.06345   3.921
##                                    Pr(>|t|)
## (Intercept)                         < 2e-16 ***
## areaBA47                           9.84e-12 ***
## gram.lexlex                         < 2e-16 ***
## sham.realsham                       < 2e-16 ***
## areaBA47:gram.lexlex                0.00501 **
## areaBA47:sham.realsham             4.94e-08 ***
## gram.lexlex:sham.realsham          6.98e-07 ***
## areaBA47:gram.lexlex:sham.realsham 8.96e-05 ***
## ---
## Signif. codes:  0 '***' 0.001 '**' 0.01 '*' 0.05 '.' 0.1 ' ' 1
##
## Correlation of Fixed Effects:
##             (Intr) arBA47 grm.lx shm.rl arBA47:g. arBA47:s. grm.:.
## areaBA47    -0.126
## gram.lexlex -0.125  0.496
## sham.relshm -0.127  0.501  0.499
## arBA47:grm.  0.089 -0.704 -0.707 -0.352
## arBA47:shm.  0.089 -0.708 -0.351 -0.705  0.499
## grm.lxlx:s.  0.089 -0.353 -0.710 -0.704  0.502     0.496
## arBA47:g.:. -0.063  0.500  0.502  0.497 -0.710    -0.706    -0.706
```

```r
# estimated marginal means
model.emmf <- emmeans(modeldur1, ~ gram.lex * area * sham.real, pbkrtest.limit = 4670)
contrast(model.emmf, "consec", simple = "each", combine = TRUE, adjust = "mvt")
```

```
##  area sham.real gram.lex contrast    estimate     SE   df t.ratio p.value
##  BA44 real      .        lex - gram    0.3111 0.0128 4629 24.315  <.0001
##  BA47 real      .        lex - gram    0.2603 0.0128 4629 20.331  <.0001
##  BA44 sham      .        lex - gram    0.2216 0.0127 4629 17.488  <.0001
##  BA47 sham      .        lex - gram    0.2708 0.0127 4629 21.251  <.0001
##  .    real      gram     BA47 - BA44   0.0870 0.0127 4629  6.826  <.0001
##  .    real      lex      BA47 - BA44   0.0362 0.0128 4629  2.820  0.0494
##  .    sham      gram     BA47 - BA44  -0.0113 0.0127 4629 -0.891  0.9711
##  .    sham      lex      BA47 - BA44   0.0378 0.0127 4629  2.981  0.0307
##  BA44 .         gram     sham - real   0.1205 0.0127 4629  9.498  <.0001
##  BA44 .         lex      sham - real   0.0311 0.0128 4629  2.430  0.1390
##  BA47 .         gram     sham - real   0.0222 0.0128 4629  1.734  0.5262
##  BA47 .         lex      sham - real   0.0327 0.0128 4629  2.560  0.1007
##
## Degrees-of-freedom method: kenward-roger
```

```
## Results are given on the log (not the response) scale.
## P value adjustment: mvt method for 12 tests
```

```r
# Dur2 as a dependent variable


# full model with random slopes
modeldur2=lmer(dur2~area*gram.lex*sham.real+(1|participant)+(1|item) + (1 + area|participant)+(1 + gram
```

```
## Warning: Model failed to converge with 3 negative eigenvalues: -1.1e-04 -1.2e-04
## -3.2e-04
```

```r
# the model didn't converge.
modeldur2=lmer(dur2~area*gram.lex*sham.real+(1|participant)+(1|item) + (1 + area|participant)+(1 + sham
```

```
## Warning in checkConv(attr(opt, "derivs"), opt$par, ctrl = control$checkConv, :
## unable to evaluate scaled gradient

## Warning in checkConv(attr(opt, "derivs"), opt$par, ctrl = control$checkConv, :
## Model failed to converge: degenerate Hessian with 1 negative eigenvalues
```

```r
modeldur2=lmer(dur2~area*gram.lex*sham.real+(1|participant)+(1|item) +(1 + sham.real|participant), data=

modeldur2=lmer(dur2~area*gram.lex*sham.real+(1|participant)+(1|item), data=target, control=lmerControl(

step(modeldur2)
```

```
## Backward reduced random-effect table:
##
##                   Eliminated npar logLik   AIC    LRT Df Pr(>Chisq)
## <none>                         11 -26445 52912
## (1 | participant)          0   10 -27767 55554 2643.6  1  < 2.2e-16 ***
## (1 | item)                 0   10 -28308 56637 3726.4  1  < 2.2e-16 ***
## ---
## Signif. codes:  0 '***' 0.001 '**' 0.01 '*' 0.05 '.' 0.1 ' ' 1
##
## Backward reduced fixed-effect table:
## Degrees of freedom method: Satterthwaite
##
##                         Eliminated Sum Sq Mean Sq NumDF  DenDF  F value
## area:gram.lex:sham.real          1   2739    2739     1 4603.1   0.5464
## area:gram.lex                    2   1143    1143     1 4604.1   0.2280
## area:sham.real                   3   2928    2928     1 4605.1   0.5843
## gram.lex:sham.real               4   7014    7014     1 4606.1   1.3998
## area                             0  40761   40761     1 4607.1   8.1335
## gram.lex                         0 869942  869942     1 4607.1 173.5908
## sham.real                        0 934017  934017     1 4607.1 186.3767
##                            Pr(>F)
## area:gram.lex:sham.real  0.459837
## area:gram.lex            0.633035
## area:sham.real           0.444656
## gram.lex:sham.real       0.236816
## area                     0.004365 **
## gram.lex                < 2.2e-16 ***
## sham.real               < 2.2e-16 ***
## ---
## Signif. codes:  0 '***' 0.001 '**' 0.01 '*' 0.05 '.' 0.1 ' ' 1
```

```
##
## Model found:
## dur2 ~ area + gram.lex + sham.real + (1 | participant) + (1 |
##     item)
```

```
# residual plots for normality check. transformation needed.
plot(fitted(modeldur2),residuals(modeldur2))
```

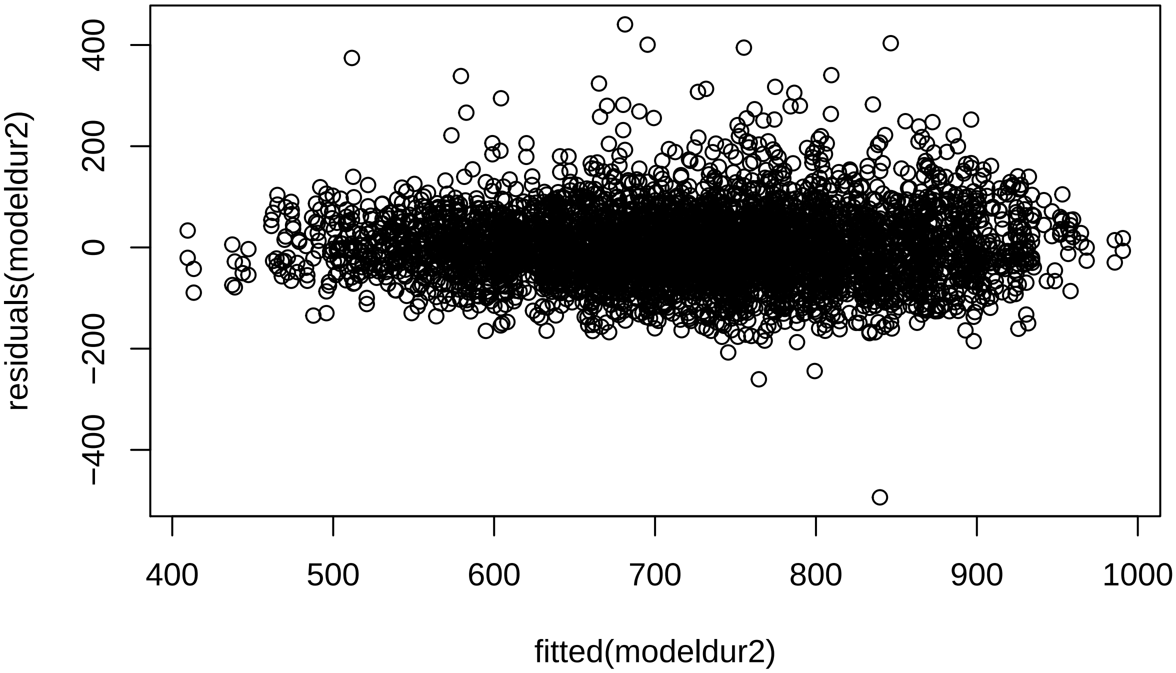


```
qqnorm(residuals(modeldur2)); qqline(residuals (modeldur2))
```

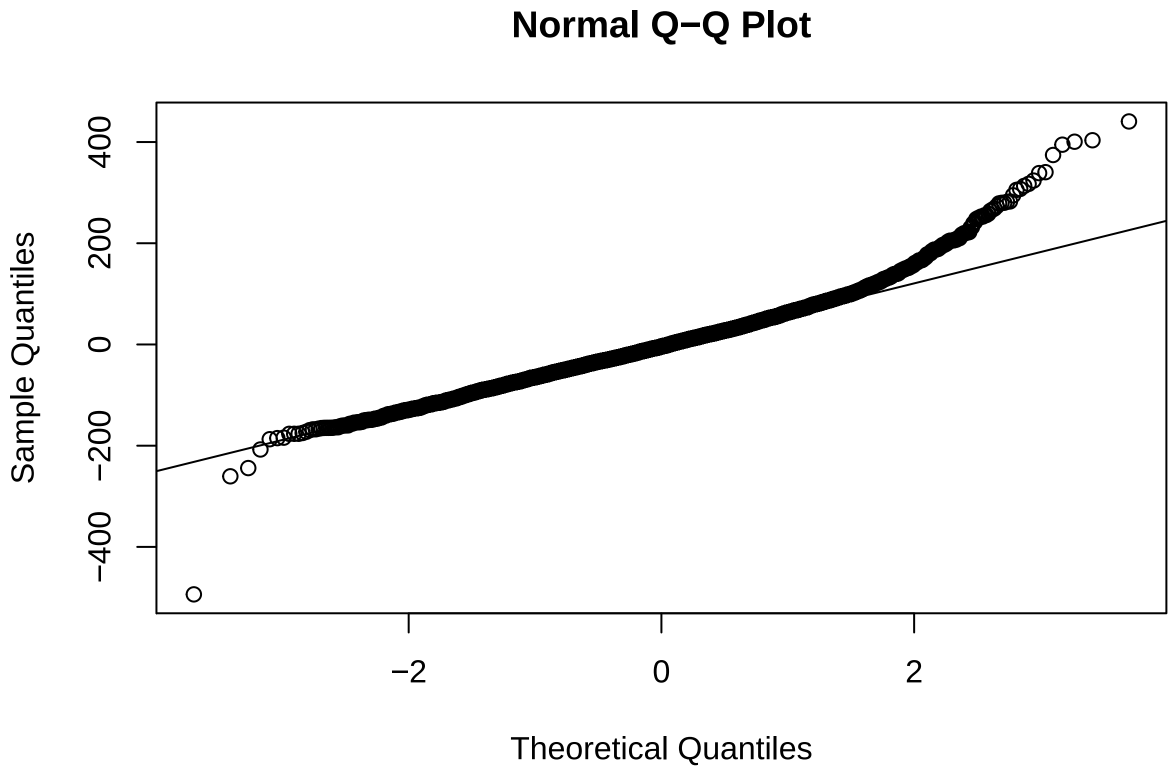

```r
# full model with random slopes
modeldur2=lmer(log(dur2)~area*gram.lex*sham.real+(1|participant)+(1|item) + (1 + area|participant)+(1 +

# the model didn't converge. continuing to remove random slopes one by one until it is converged.
modeldur2=lmer(log(dur2)~area*gram.lex*sham.real+(1|participant)+(1|item) + (1 + area|participant)+(1 +
```

```
## Warning in checkConv(attr(opt, "derivs"), opt$par, ctrl = control$checkConv, :
## unable to evaluate scaled gradient

## Warning in checkConv(attr(opt, "derivs"), opt$par, ctrl = control$checkConv, :
## Model failed to converge: degenerate Hessian with 1 negative eigenvalues

## Warning: Model failed to converge with 2 negative eigenvalues: -8.4e-05 -6.6e-04
```

```r
modeldur2=lmer(log(dur2)~area*gram.lex*sham.real+(1|participant)+(1|item) +(1 + sham.real|participant),
modeldur2=lmer(log(dur2)~area*gram.lex*sham.real+(1|participant)+(1|item), data=target, control=lmerCon
step(modeldur2)
```

```
## Backward reduced random-effect table:
##
##                     Eliminated npar logLik     AIC    LRT Df Pr(>Chisq)
## <none>                           11 4105.2 -8188.4
## (1 | participant)          0   10 2702.2 -5384.4 2806.1  1  < 2.2e-16 ***
## (1 | item)                 0   10 2157.1 -4294.3 3896.2  1  < 2.2e-16 ***
## ---
## Signif. codes:  0 '***' 0.001 '**' 0.01 '*' 0.05 '.' 0.1 ' ' 1
##
## Backward reduced fixed-effect table:
## Degrees of freedom method: Satterthwaite
##
##                          Eliminated  Sum Sq Mean Sq NumDF  DenDF  F value
## area:gram.lex:sham.real          1 0.00880 0.00880     1 4603.1   0.9302
## area:gram.lex                    2 0.00228 0.00228     1 4604.1   0.2405
## area:sham.real                   3 0.01039 0.01039     1 4605.1   1.0981
## gram.lex:sham.real               4 0.03450 0.03450     1 4606.1   3.6468
## area                             0 0.09319 0.09319     1 4607.1   9.8443
## gram.lex                         0 1.62248 1.62248     1 4607.1 171.3920
## sham.real                        0 1.74788 1.74788     1 4607.1 184.6388
##                            Pr(>F)
## area:gram.lex:sham.real  0.334874
## area:gram.lex            0.623901
## area:sham.real           0.294743
## gram.lex:sham.real       0.056239 .
## area                     0.001714 **
## gram.lex                < 2.2e-16 ***
## sham.real               < 2.2e-16 ***
## ---
## Signif. codes:  0 '***' 0.001 '**' 0.01 '*' 0.05 '.' 0.1 ' ' 1
##
## Model found:
## log(dur2) ~ area + gram.lex + sham.real + (1 | participant) +
##     (1 | item)
```

```r
# the model suggested by the step function.
modeldur2 = lmer(log(dur2) ~ area + gram.lex + sham.real + (1 | participant) + (1 |
```

```r
    item), data = target, control=lmerControl(optimizer = "bobyqa", optCtrl=list(maxfun=100000)))
summary(modeldur2)
```

```
## Linear mixed model fit by REML. t-tests use Satterthwaite's method [
## lmerModLmerTest]
## Formula: log(dur2) ~ area + gram.lex + sham.real + (1 | participant) +
##     (1 | item)
##    Data: target
## Control: lmerControl(optimizer = "bobyqa", optCtrl = list(maxfun = 1e+05))
##
## REML criterion at convergence: -8237.1
##
## Scaled residuals:
##     Min      1Q  Median      3Q     Max
## -9.0918 -0.6153 -0.0159  0.5839  5.6371
##
## Random effects:
##  Groups      Name        Variance Std.Dev.
##  participant (Intercept) 0.008536 0.09239
##  item        (Intercept) 0.013804 0.11749
##  Residual                0.009466 0.09730
## Number of obs: 4644, groups:  participant, 19; item, 16
##
## Fixed effects:
##                 Estimate Std. Error        df t value Pr(>|t|)
## (Intercept)    6.572e+00  3.633e-02  2.849e+01 180.865  < 2e-16 ***
## areaBA47      -8.966e-03  2.858e-03  4.607e+03  -3.138  0.00171 **
## gram.lexlex   -3.742e-02  2.858e-03  4.607e+03 -13.092  < 2e-16 ***
## sham.realsham  3.885e-02  2.859e-03  4.607e+03  13.588  < 2e-16 ***
## ---
## Signif. codes:  0 '***' 0.001 '**' 0.01 '*' 0.05 '.' 0.1 ' ' 1
##
## Correlation of Fixed Effects:
##             (Intr) arBA47 grm.lx
## areaBA47    -0.039
## gram.lexlex -0.039  0.003
## sham.relshm -0.039 -0.004 -0.011
```

```r
# estimated marginal means
model.emmf <- emmeans(modeldur2, ~ gram.lex + area + sham.real, pbkrtest.limit = 4670)
contrast(model.emmf, "consec", simple = "each", combine = TRUE, adjust = "mvt")
```

```
##  area sham.real gram.lex contrast    estimate      SE   df t.ratio p.value
##  BA44 real      .        lex - gram  -0.03742 0.00286 4607 -13.092 <.0001
##  BA47 real      .        lex - gram  -0.03742 0.00286 4607 -13.092 <.0001
##  BA44 sham      .        lex - gram  -0.03742 0.00286 4607 -13.092 <.0001
##  BA47 sham      .        lex - gram  -0.03742 0.00286 4607 -13.092 <.0001
##  .    real      gram     BA47 - BA44 -0.00897 0.00286 4607  -3.138 0.0051
##  .    real      lex      BA47 - BA44 -0.00897 0.00286 4607  -3.138 0.0051
##  .    sham      gram     BA47 - BA44 -0.00897 0.00286 4607  -3.138 0.0051
##  .    sham      lex      BA47 - BA44 -0.00897 0.00286 4607  -3.138 0.0051
##  BA44 .         gram     sham - real  0.03885 0.00286 4607  13.588 <.0001
##  BA44 .         lex      sham - real  0.03885 0.00286 4607  13.588 <.0001
##  BA47 .         gram     sham - real  0.03885 0.00286 4607  13.588 <.0001
```

```
##  BA47 .         lex      sham - real  0.03885 0.00286 4607  13.588 <.0001
##
## Degrees-of-freedom method: kenward-roger
## Results are given on the log (not the response) scale.
## P value adjustment: mvt method for 12 tests
```

```r
## Bar plots with three factors (Figure 3 in the manuscript)
library(ggplot2)
library(ggpubr)
```

```
## Loading required package: magrittr
```

```
##
## Attaching package: 'magrittr'
```

```
## The following object is masked from 'package:pastecs':
##
##     extract
```

```
## The following object is masked from 'package:purrr':
##
##     set_names
```

```
## The following object is masked from 'package:tidyr':
##
##     extract
```

```r
data = target
data$area = gsub("BA44", "pIFG", data$area)
data$area = gsub("BA47", "aIFG", data$area)
data$Stimulation = data$sham.real
data$Stimulation = gsub("real", "effective", data$sham.real)
data$gram.lex = gsub("lex", "Lexical task", data$gram.lex)
data$gram.lex = gsub("gram", "Grammatical task", data$gram.lex)
data$gram.lex = as.factor(data$gram.lex)
data$sham.real = as.factor(data$sham.real)
data$area = as.factor(data$area)

# dataframes with the coordinates for the asterisk and geom_segment for RTs
anno4 <- data.frame (x5 = 0, x6 = 2, y5 = 1075, y6 = 1100, xstar = 0, ystar = 1125, lab = c("", "*"), sl
anno3 <- data.frame (x5 = 1, x6 = 3, y5 = 1075, y6 = 1100, xstar = 3, ystar = 1125, lab = c("*", ""), sl


# plotting RTs
bar1 = ggplot(data, aes(x=area, y=rt, group=sham.real))
p1<-bar1  + stat_summary(fun.y=mean, geom="bar", aes(fill=sham.real), position = position_dodge())+
  stat_summary(fun.data=mean_cl_boot, geom="errorbar", width=0.08, position = position_dodge(0.9)) +
  facet_wrap(~gram.lex)+
  scale_fill_manual(values=c("darkred", "blue"))+
  guides(fill=guide_legend(title="Stimulation"))+
  labs(x="Target site", y="RTs ± SEM (ms)")+
  theme_bw() + theme(panel.spacing.x=unit(0, "lines"), panel.border = element_blank(), panel.grid.major
                     panel.grid.minor = element_blank(), axis.line = element_line(colour = "black"),
                     axis.text.x = element_text(size=18), axis.text.y = element_text(size=18),
                     axis.title=element_text(size=18),
                     strip.text = element_text(size=18),
                     legend.position = "none")+expand_limits(y=1200)+
```

```
   theme(strip.background =element_blank())+
  geom_text(x = 1,  y = 925,
           label = "***",
           colour = "black", size = 8) +
  geom_segment(x = 0.75, xend = 0.75,
              y = 875, yend = 900,
              colour = "black") +
  geom_segment(x = 0.75, xend = 1.25,
              y = 900, yend = 900,
              colour = "black") +
  geom_segment(x = 1.25, xend = 1.25,
              y = 900, yend = 875,
              colour = "black")+
  geom_text(x = 1.5,  y = 1025,
           label = "***",
           colour = "black", size = 8)+
  geom_segment(x = 1, xend = 1,
              y = 975, yend = 1000,
              colour = "black") +
  geom_segment(x = 1, xend = 2,
              y = 1000, yend = 1000,
              colour = "black") +
  geom_segment(x = 2, xend = 2,
              y = 1000, yend = 975,
              colour = "black")+
  geom_text(data = anno3, aes(x = xstar,  y = ystar,
                             label = lab), size = 8,
           colour = "black")+
  geom_text(data = anno4, aes(x = xstar,  y = ystar,
                              label = lab), size = 8,
           colour = "black")+
  geom_segment(data = anno3, aes(x = x5, xend = x5,
                                 y = y5, yend = y6),
              colour = "black") +
  geom_segment(data = anno4, aes (x = x5, xend = x6,
                                  y = y6, yend = y6),
              colour = "black")+
  geom_segment(data = anno3, aes(x = x5, xend = x6,
                                  y = y6, yend = y6),
              colour = "black") +
  geom_segment(data = anno4, aes (x = x6, xend = x6,
                                   y = y5, yend = y6),
              colour = "black")+
  scale_y_continuous(breaks = seq(0, 1200, 200), labels = seq(0, 1200, 200))

p1
```

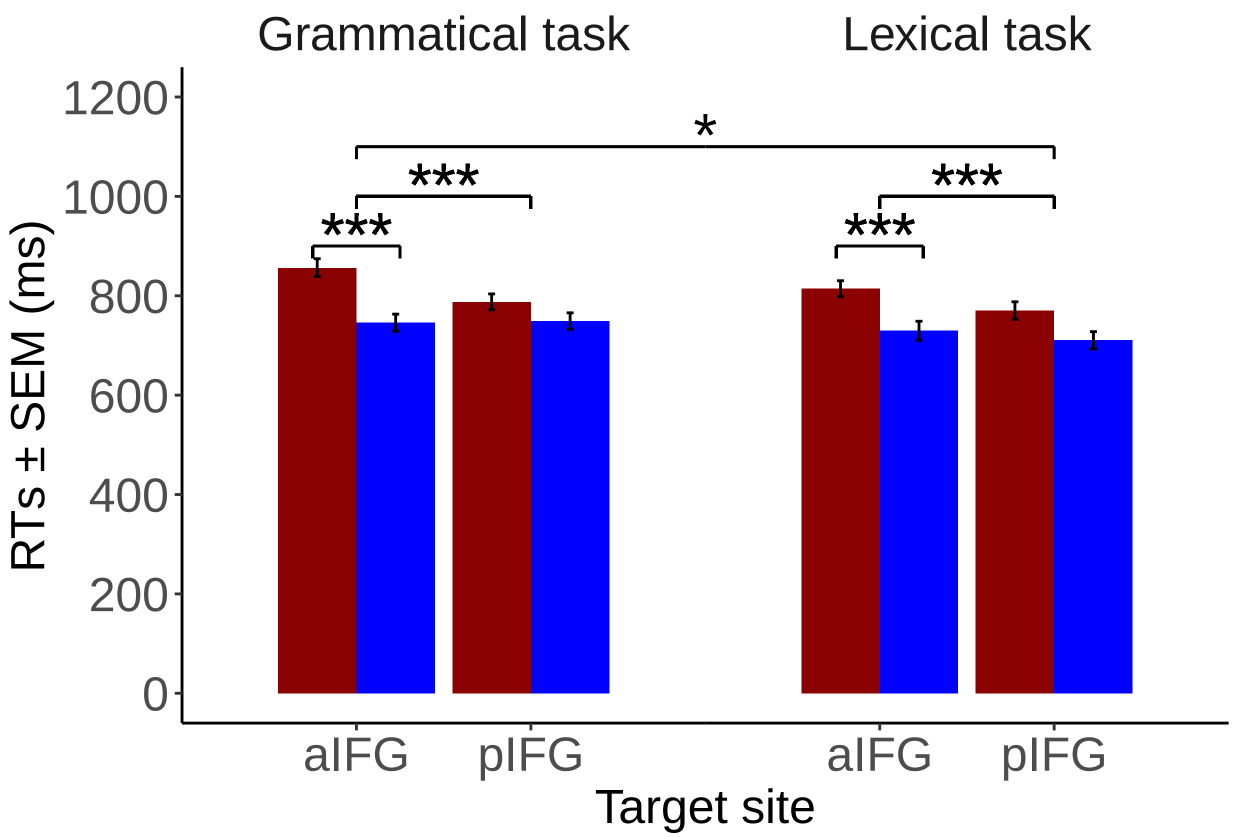


```
# creating a dataframe with coordinates for asterisk and geom_segment to plot dur 1
anno <- data.frame (x1 = 1.75, x2 = 2.25, y1 = 200, y2 = 225, x3 = 1, x4 =  2, y3 = 275, y4 = 300, x5 =
                    xstar2 = 1.5, ystar2 = 325, xstar3 = 3, ystar3 = 425, lab = "***", sham.real= c("ef

anno2 <- data.frame (x5 = 0, x6 = 2, y5 = 375, y6 = 400, xstar = 0, ystar = 425, lab = "***", sham.real

# plotting dur 1

bar2 = ggplot(data = data, aes(x=area, y=dur1, group=sham.real))
p2<-bar2  + stat_summary(fun.y=mean, geom="bar", aes(fill=sham.real), position = position_dodge())+
  stat_summary(fun.data=mean_cl_boot, geom="errorbar", width=0.08, position = position_dodge(0.9)) +
  facet_wrap(~gram.lex)+
  scale_fill_manual(values=c("darkred", "blue"))+
  guides(fill=guide_legend(title="Stimulation"))+
  labs(x="Target site", y="Dur 1 ± SEM (ms)")+
  theme_bw() + theme(panel.spacing.x=unit(0, "lines"), panel.border = element_blank(), panel.grid.major
                     panel.grid.minor = element_blank(), axis.line = element_line(colour = "black"),
                     axis.text.x = element_text(size=18), axis.text.y = element_text(size=18),
                     axis.title=element_text(size=18),
                     strip.text = element_text(size=18),
                     legend.position = "none")+expand_limits(y=1200)+
  theme(strip.background =element_blank())+
  geom_text(data = anno, aes(x = xstar,  y = ystar,
            label = lab),inherit.aes = FALSE, size = 8,
            colour = "black") +
  geom_segment(data = anno, aes(x = x1, xend = x1,
               y = y1, yend = y2),
```

```
               colour = "black") +
  geom_segment(data = anno, aes(x = x1, xend = x2,
               y = y2, yend = y2),
               colour = "black") +
  geom_segment(data = anno, aes(x = x2, xend = x2,
               y = y1, yend = y2),
               colour = "black")+
  geom_text(data = anno, aes(x = xstar2,  y = ystar2,
            label = "***"), size = 8,
            colour = "black") +
  geom_segment(data = anno, aes(x = x3, xend = x3,
               y = y3, yend = y4),
               colour = "black") +
  geom_segment(data = anno, aes(x = x3, xend = x4,
               y = y4, yend = y4),
               colour = "black") +
  geom_segment(data = anno, aes (x = x4, xend = x4,
               y = y3, yend = y4),
               colour = "black")+
  geom_text(data = anno, aes(x = xstar3,  y = ystar3,
            label = lab), size = 8,
            colour = "black")+
  geom_text(data = anno2, aes(x = xstar,  y = ystar,
                              label = lab), size = 8,
            colour = "black")+
  geom_segment(data = anno, aes(x = x5, xend = x5,
                                y = y5, yend = y6),
               colour = "black") +
  geom_segment(data = anno, aes (x = x5, xend = x6,
                                 y = y6, yend = y6),
               colour = "black")+
  geom_segment(data = anno2, aes(x = x5, xend = x6,
                                y = y6, yend = y6),
               colour = "black") +
  geom_segment(data = anno2, aes (x = x6, xend = x6,
                                 y = y5, yend = y6),
               colour = "black")+
  scale_y_continuous(breaks = seq(0, 1200, 200), labels = seq(0, 1200, 200))

p2
```

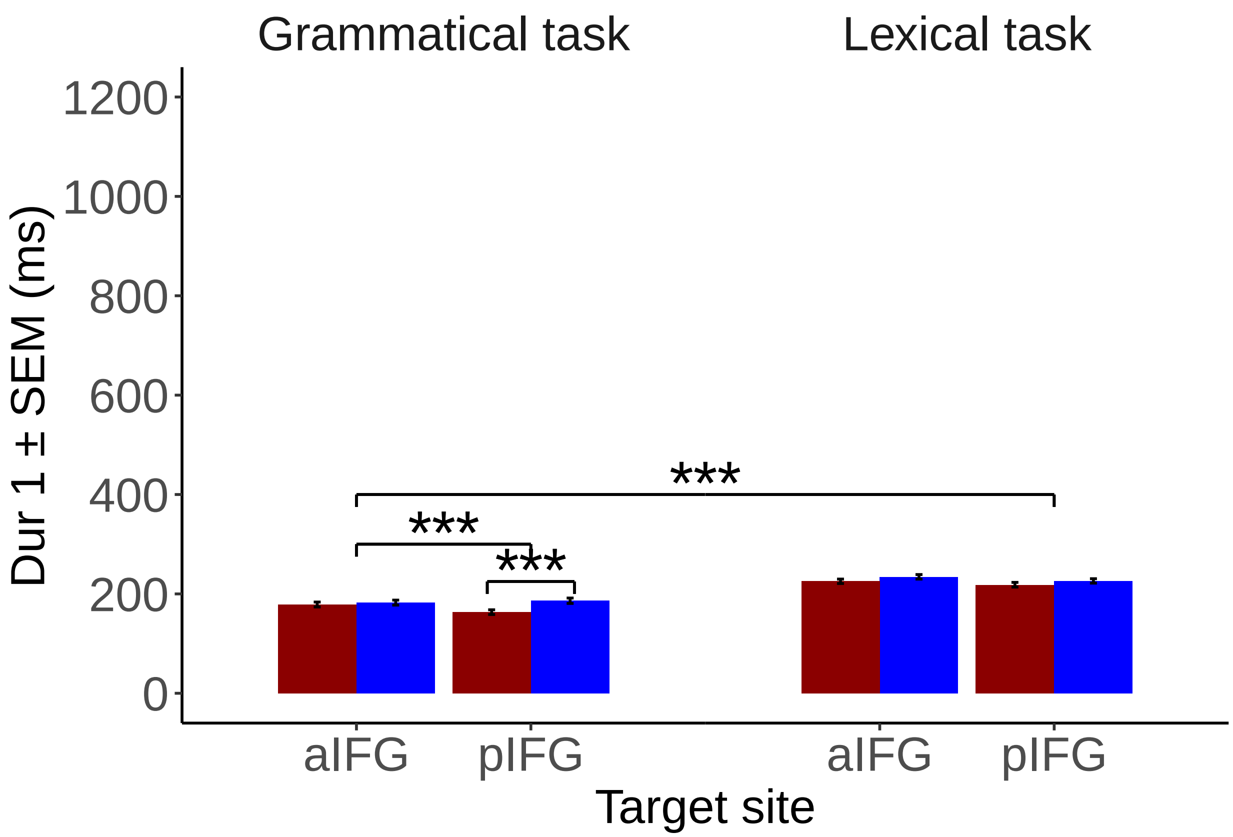


```
# plotting dur 2

# creating a dataframe with coordinates for asterisk and geom_segment to plot dur 1
anno <- data.frame (x1 = 0.75, x2 = 2.25, y1 = 775, y2 = 825, x3 = 1, x4 =  2, y3 = 275, y4 = 300, x5 =
                    xstar2 = 1.5, ystar2 = 325, xstar3 = 3, ystar3 = 425, lab = "***", sham.real= c("ef

anno2 <- data.frame (x5 = 0, x6 = 2, y5 = 375, y6 = 400, xstar = 0, ystar = 425, lab = "***", sham.real


anno44 <- data.frame (x5 = 0, x6 = 2, y5 = 975, y6 = 1000, xstar = 0, ystar = 1025, lab = c("***", "***
anno33 <- data.frame (x5 = 1, x6 = 3, y5 = 975, y6 = 1000, xstar = 3, ystar = 1025, lab = c("***", "***

bar3 = ggplot(data, aes(x=area, y=dur2, group=sham.real))
p3<-bar3  + stat_summary(fun.y=mean, geom="bar", aes(fill=sham.real), position = position_dodge())+face
  stat_summary(fun.data=mean_cl_boot, geom="errorbar", width=0.08, position = position_dodge(0.9)) +
  scale_fill_manual(values=c("darkred", "blue"))+
  guides(fill=guide_legend(title="Stimulation"))+
  labs(x="Target site", y="Dur 2 ± SEM (ms)")+
  theme_bw() + theme(panel.spacing.x=unit(0, "lines"), panel.border = element_blank(), panel.grid.major
                     panel.grid.minor = element_blank(), axis.line = element_line(colour = "black"),
                     axis.text.x = element_text(size=18), axis.text.y = element_text(size=18),
                     axis.title=element_text(size=18),legend.text = element_text(size=18),
                     strip.text = element_text(size=18),
                     legend.title = element_text(size=18))+expand_limits(y=1200)+
  theme(strip.background =element_blank())+
  geom_text(x = 1,  y = 825,
            label = "***", size = 8,
```

```
            colour = "black") +
  geom_segment(x = 0.75, xend = 0.75,
               y = 775, yend = 800,
               colour = "black") +
  geom_segment(x = 0.75, xend = 1.25,
               y = 800, yend = 800,
               colour = "black") +
  geom_segment(x = 1.25, xend = 1.25,
               y = 800, yend = 775,
               colour = "black")+
  geom_text(x = 2,  y = 825,
            label = "***", size = 8,
            colour = "black") +
  geom_segment(x = 1.75, xend = 1.75,
               y = 775, yend = 800,
               colour = "black") +
  geom_segment(x = 1.75, xend = 2.25,
               y = 800, yend = 800,
               colour = "black") +
  geom_segment(x = 2.25, xend = 2.25,
               y = 800, yend = 775,
               colour = "black")+
  geom_text(data = anno33, aes(x = xstar,  y = ystar,
                               label = lab), size = 8,
            colour = "black")+
  geom_text(data = anno44, aes(x = xstar,  y = ystar,
                                label = lab), size = 8,
            colour = "black")+
  geom_segment(data = anno33, aes(x = x5, xend = x5,
                                  y = y5, yend = y6),
               colour = "black") +
  geom_segment(data = anno44, aes (x = x5, xend = x6,
                                   y = y6, yend = y6),
               colour = "black")+
  geom_segment(data = anno33, aes(x = x5, xend = x6,
                                   y = y6, yend = y6),
               colour = "black") +
  geom_segment(data = anno44, aes (x = x6, xend = x6,
                                    y = y5, yend = y6),
               colour = "black")+
scale_y_continuous(breaks = seq(0, 1200, 200), labels = seq(0, 1200, 200))

p3
```

```
## Warning: Removed 26 rows containing non-finite values (stat_summary).

## Warning: Removed 26 rows containing non-finite values (stat_summary).
```

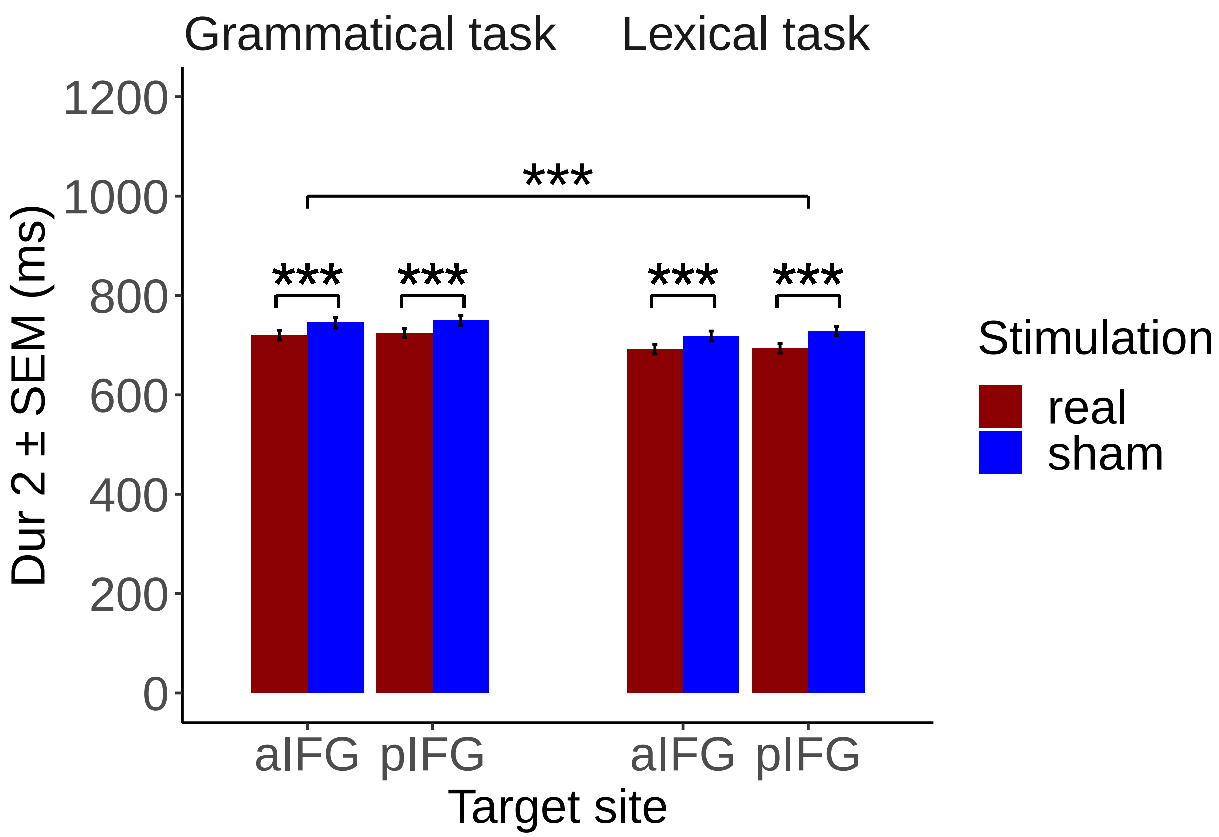


```r
#p <- ggarrange(p1, p2, p3, ncol = 3, labels = c("A", "B", "C"))
```

```r
# Loading the delta values (sham - effective)
setwd("~/Documents/DataR")
d = read.csv("tms_deltas.csv", sep = ";")
tradeoff=lm(rtn~dur1n, data=d)
summary(tradeoff)
```

```
##
## Call:
## lm(formula = rtn ~ dur1n, data = d)
##
## Residuals:
##     Min      1Q  Median      3Q     Max
## -369.88  -85.40  -18.96  105.40  355.50
##
## Coefficients:
##             Estimate Std. Error t value Pr(>|t|)
## (Intercept) -86.7046    17.1150  -5.066 2.89e-06 ***
## dur1n         1.3068     0.3765   3.471 0.000869 ***
## ---
## Signif. codes:  0 '***' 0.001 '**' 0.01 '*' 0.05 '.' 0.1 ' ' 1
##
## Residual standard error: 145.1 on 74 degrees of freedom
## Multiple R-squared:   0.14,  Adjusted R-squared:  0.1284
## F-statistic: 12.05 on 1 and 74 DF,  p-value: 0.0008694
```

```
tradeoff2=lm(rtn~dur2n, data=d)
summary(tradeoff2)
```

```
##
## Call:
## lm(formula = rtn ~ dur2n, data = d)
##
## Residuals:
##      Min       1Q   Median       3Q      Max
## -304.020 -103.720   -6.387  118.055  283.188
##
## Coefficients:
##              Estimate Std. Error t value Pr(>|t|)
## (Intercept) -108.0922    17.5051  -6.175 3.25e-08 ***
## dur2n          1.2826     0.2756   4.654 1.40e-05 ***
## ---
## Signif. codes:  0 '***' 0.001 '**' 0.01 '*' 0.05 '.' 0.1 ' ' 1
##
## Residual standard error: 137.7 on 74 degrees of freedom
## Multiple R-squared:  0.2264, Adjusted R-squared:  0.2159
## F-statistic: 21.66 on 1 and 74 DF,  p-value: 1.4e-05
```

```
# Plotting the dependent variables
ggplot(d, aes(x=rtn, y=dur1n)) + geom_point(shape=1) +
  scale_colour_hue(l=50) +  geom_smooth(method=lm, se=TRUE, fullrange=TRUE)+labs(x=expression(Delta~"RTs
  theme_bw() + theme(panel.border = element_blank(), panel.grid.major = element_blank(),
                     panel.grid.minor = element_blank(), axis.line = element_line(colour = "black"),axis
```

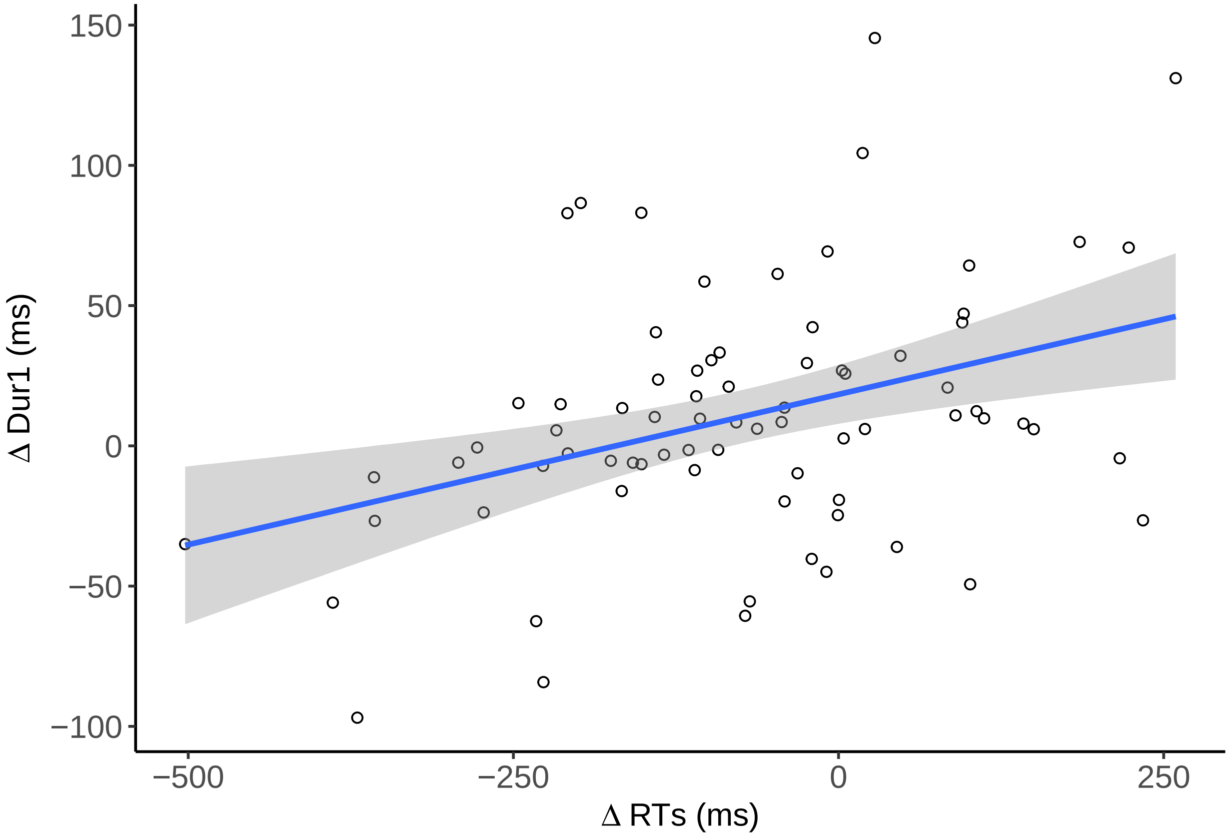

```
ggplot(d, aes(x=rtn, y=dur2n)) + geom_point(shape=1) +
  scale_colour_hue(l=50) +  geom_smooth(method=lm, se=TRUE, fullrange=TRUE)+labs(x=expression(Delta~"RT
```

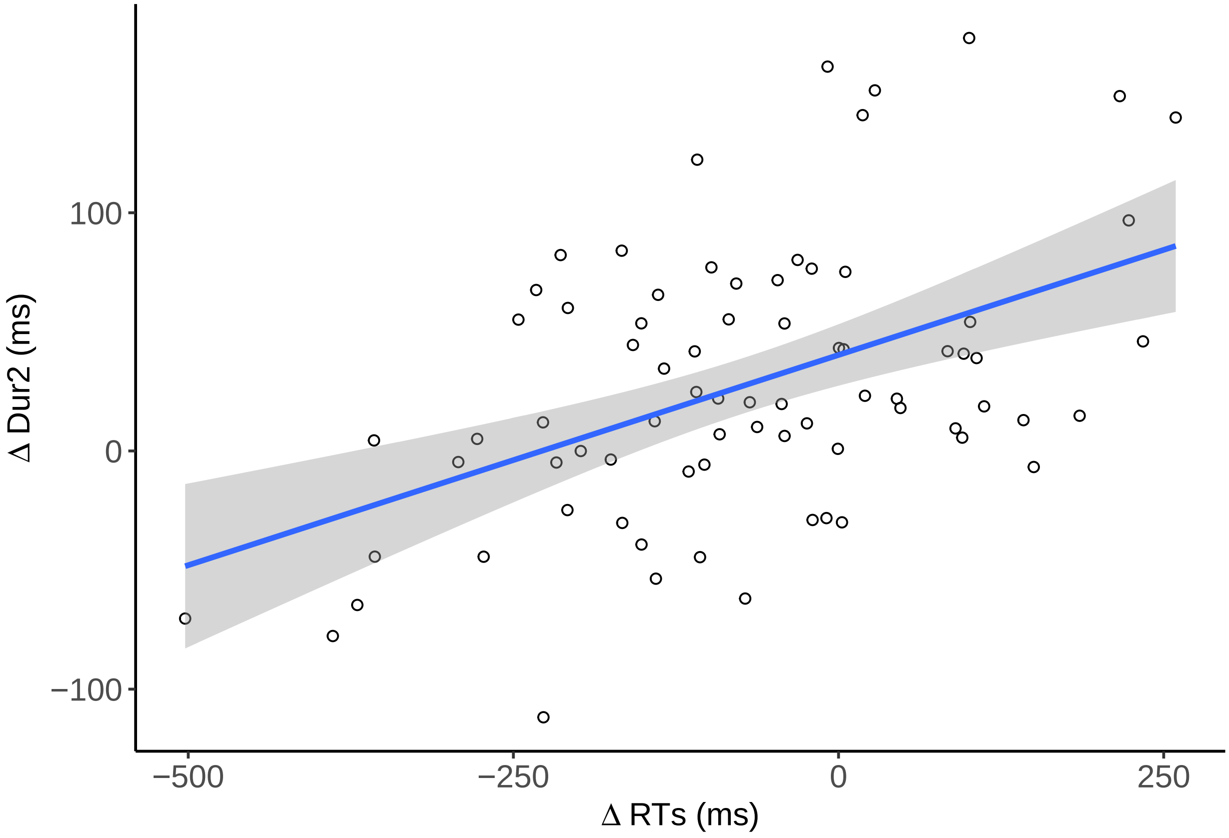

Supplement: Supplementary file 1 [file Data_Sheet_1.pdf]
